# Targeting isoaspartate-modified Aβ rescues behavioral deficits in transgenic mice with Alzheimer’s disease-like pathology

**DOI:** 10.1186/s13195-020-00719-x

**Published:** 2020-11-14

**Authors:** Kathrin Gnoth, Anke Piechotta, Martin Kleinschmidt, Sandra Konrath, Mathias Schenk, Nadine Taudte, Daniel Ramsbeck, Vera Rieckmann, Stefanie Geissler, Rico Eichentopf, Susan Barendrecht, Maike Hartlage-Rübsamen, Hans-Ulrich Demuth, Steffen Roßner, Holger Cynis, Jens-Ulrich Rahfeld, Stephan Schilling

**Affiliations:** 1 Department of Drug Design and Target Validation, Fraunhofer Institute for Cell Therapy and Immunology, Halle (Saale), Germany; 2 Present address: Institute of Clinical Chemistry and Laboratory Medicine, University Medical Center Hamburg-Eppendorf, Hamburg, Germany; 3 Present address: PerioTrap Pharmaceuticals GmbH, Halle (Saale), Germany; 4 Present address: Fraunhofer Center for Chemical-Biotechnological Processes CBP, Leuna, Germany; 5 Paul Flechsig Institute of Brain Research, Leipzig University, Leipzig, Germany

**Keywords:** Passive immunotherapy, Isoaspartate, Amyloid beta, 5xFAD mouse model, Alzheimer’s disease

## Abstract

**Background:**

Amyloid β (Aβ)-directed immunotherapy has shown promising results in preclinical and early clinical Alzheimer’s disease (AD) trials, but successful translation to late clinics has failed so far. Compelling evidence suggests that post-translationally modified Aβ peptides might play a decisive role in onset and progression of AD and first clinical trials targeting such Aβ variants have been initiated. Modified Aβ represents a small fraction of deposited material in plaques compared to pan-Aβ epitopes, opening up pathways for tailored approaches of immunotherapy. Here, we generated the first monoclonal antibodies that recognize l-isoaspartate-modified Aβ (isoD7-Aβ) and tested a lead antibody molecule in 5xFAD mice.

**Methods:**

This work comprises a combination of chemical and biochemical techniques as well as behavioral analyses. Aβ peptides, containing l-isoaspartate at position 7, were chemically synthesized and used for immunization of mice and antibody screening methods. Biochemical methods included anti-isoD7-Aβ monoclonal antibody characterization by surface plasmon resonance, immunohistochemical staining of human and transgenic mouse brain, and the development and application of isoD7-Aβ ELISA as well as different non-modified Aβ ELISA. For antibody treatment studies, 12 mg/kg anti-isoD7-Aβ antibody K11_IgG2a was applied intraperitoneally to 5xFAD mice for 38 weeks. Treatment controls implemented were IgG2a isotype as negative and 3D6_IgG2a, the parent molecule of bapineuzumab, as positive control antibodies. Behavioral studies included elevated plus maze, pole test, and Morris water maze.

**Results:**

Our advanced antibody K11 showed a K_D_ in the low nM range and > 400fold selectivity for isoD7-Aβ compared to other Aβ variants. By using this antibody, we demonstrated that formation of isoD7-Aβ may occur after formation of aggregates; hence, the presence of the isoD7-modification differentiates aged Aβ from newly formed peptides. Importantly, we also show that the Tottori mutation responsible for early-onset AD in a Japanese pedigree is characterized by massively accelerated formation of isoD7-Aβ in cell culture. The presence of isoD7-Aβ was verified by K11 in post mortem human cortex and 5xFAD mouse brain tissue. Passive immunization of 5xFAD mice resulted in a significant reduction of isoD7-Aβ and total Aβ in brain. Amelioration of cognitive impairment was demonstrated by Morris water maze, elevated plus maze, pole, and contextual fear conditioning tests. Interestingly, despite the lower abundance of the isoD7-Aβ epitope, the application of anti-isoD7-Aβ antibodies showed comparable treatment efficacy in terms of reduction of brain amyloid and spatial learning but did not result in an increase of plasma Aβ concentration as observed with 3D6 treatment.

**Conclusions:**

The present study demonstrates, for the first time, that the antibody-mediated targeting of isoD7-modified Aβ peptides leads to attenuation of AD-like amyloid pathology. In conjunction with previously published data on antibodies directed against pGlu-modified Aβ, the results highlight the crucial role of modified Aβ peptides in AD pathophysiology. Hence, the results also underscore the therapeutic potential of targeting modified amyloid species for defining tailored approaches in AD therapy.

**Supplementary information:**

The online version contains supplementary material available at 10.1186/s13195-020-00719-x.

## Background

Alzheimer’s disease (AD) is a progressive, incurable neurodegenerative disorder, occurring in mid to late life. The consequences of AD always lead to death, usually 7 to 10 years after diagnosis [1]. Currently, 40 million people are affected, which makes AD the most common neurodegenerative disorder worldwide [2].

Two main histological alterations can be discerned in the post mortem brain of AD patients: extracellular senile plaques [3] and intracellular neurofibrillary tangles [4]. The former are basically composed of fibrillar Aβ. Aβ peptides are formed by endoproteolytic cleavage of amyloid precursor protein (APP) [5]. A number of specific mutations in genes related to Aβ production ultimately result in the development of AD. This provides strong support for the amyloid hypothesis of AD, stating that accumulating Aβ represents the central trigger for a cascade of pathological brain changes, eliciting tau hyperphosphorylation, neuronal damage, synapse and cell loss, and dementia [6]. However, it remains unresolved how Aβ exerts its toxic effects and numerous drug failures in the past, among those small molecule inhibitors of Aβ production and monoclonal antibodies, call the amyloid hypothesis into question. Compelling evidence now also suggests that post-translational modifications of Aβ peptides accelerate their aggregation and induce toxicity, in turn driving disease progression [7–11]. One of these post-translational modifications is the formation of isoaspartate (isoD). The generation of isoD from l-asparaginyl or l-aspartyl residues was extensively described earlier [12, 13]. Both residues can isomerize spontaneously via an l-succinimidyl intermediate, which either hydrolyzes into l-isoaspartate (in 2/3 of the cases) or l-aspartate (in 1/3 of the cases). A minor proportion of l-succinimide might also epimerize to d-succinimide at a slow rate, leading to the formation of d-isoaspartate and d-aspartate residues, respectively. The isomerization of asparagine and aspartate residues is a spontaneous post-translational modification, which is considered to determine the half-life of proteins [14–16]. Moreover, isoD formation introduces an additional methylene group into the backbone of the protein or peptide [17, 18], consequently altering its structure. Hence, this modification may also change the properties of proteins like solubility, conformation, and function. The presence of isoD7-Aβ variants in brain of AD patients was first described in 1993 [12]. By using polyclonal anti-isoD7-Aβ antibodies, it was shown that isoD7-Aβ is present in extracellular deposits in AD brain as well as amyloid-bearing vessels and serves as an indicator of plaque age [13, 19].

The influence of isoD7-Aβ on amyloid plaque formation is controversially discussed. Despite the fact that isoD7 modification might not influence aggregation of the Aβ peptide [10, 20], it may be involved in the onset of AD. This modification contributes to the insolubility and stability of Aβ [21], is located within the zinc-binding site of Aβ [22], and was described to influence zinc-dependent oligomerization of Aβ (1–16) monomers [23] as well as hydrolysis of Aβ by the angiotensin-converting enzyme [24]. Furthermore, isoD7-Aβ was shown to be an exogenous trigger of extensive amyloid plaque formation in AD models [25, 26] and isoD7-Aβ (1–42) is more toxic for neuronal cells than non-modified Aβ (1–42) [27]. Finally, further evidence for an involvement of isoD7-Aβ in AD pathology results from an inherited form of AD called Japanese-Tottori FAD. In the affected members of this family, a missense mutation within APP (D678N) replaces the aspartate 7 of Aβ with asparagine [28]. Asparagine residues undergo about 10 times more rapidly isomerization than aspartate [29, 30]. Manifestation of AD symptoms in this pedigree may not be due to N7-Aβ, but to the enhanced formation of isoD7-Aβ.

On our quest to decipher the role of posttranslational modifications of peptides and proteins in protein misfolding disorders, we here aimed at investigating the isoD7-Aβ modification. To achieve this goal, we generated specific antibodies recognizing isoD7-Aβ. These antibodies were then used to study the formation of isoD7 in Aβ in vitro and in vivo. Furthermore, we applied one of the antibodies to 5xFAD mice to study a potential therapeutic effect of removing isoD7-Aβ from transgenic mouse brain. The results strongly imply an accumulation of isoD7-Aβ with progression of pathology. A specific targeting of isoD7-Aβ might thus represent a therapeutic strategy for treatment of AD.

## Methods

### Generation of isoD7-Aβ peptides

The synthesis of the peptides was performed according to standard Fmoc solid phase peptide synthesis on a Tetras peptide synthesizer (Advanced ChemTech, Louisville, USA). The C- and N-truncated Aβ-peptides were synthesized at 60-μmol scale as C-terminal amides on Rink amide resin (Iris Biotech) using standard Fmoc/tBu-protected amino acids (Iris Biotech). Non-canonical amino acids were incorporated using Fmoc-L-Asp-OtBu (isoD), Fmoc-D-Asp-OtBu (isod), Fmoc-Tyr (3NO2)-OH (3NY), Fmoc-Ser (PO (OBzl)OH)-OH (phosphoSer), and Boc-Pyr-OH (pE) (Merck Millipore, Iris Biotech, Bachem). The full-length Aβ1–40 peptides were synthesized at 60-μmol scale as C-terminal acids on Fmoc-Val-Novasyn TGA resin (Merck Millipore). Coupling was performed using O-(benzotriazol-1-yl)-*N*,*N*,*N*′,*N*′-tetramethyluronium tetrafluoroborate (TBTU) and *N*-methylmorpholine (NMM). Fmoc-deprotection was carried out using 20% piperidine in DMF. Final cleavage and deprotection of the peptides was performed using TFA:DOTA (or EDT):H_2_O:TIS (30:2:2:1 v/v). After precipitation with cold diethylether, the peptides were purified by preparative RP-HPLC (Phenomenex Luna C18 (2) column, eluents: water and acetonitrile containing 0.04% TFA). Purity and identity were assessed by analytical RP-HPLC, MALDI-TOF MS or ESI MS. However, during the synthesis of isoD7-Aβ (1–40) predominant formation of succinic imide side product was detected by MALDI-MS, which was assigned to a cyclization of the isoD side chain. Hence, in this case, the isoD-residue was introduced via a Fmoc-isoD-Ser-OH pseudoproline dipeptide building block [31], which was synthesized prior to peptide synthesis by coupling Fmoc-Asp-OtBu and H-Ser-OH using NHS/EDC followed by subsequent cyclization by means of 2,2-dimethoxypropane and p-toluenesulfonic acid.

#### Antibody derivation, generation, and biophysical characterization

The antibody 3D6 with IgG2b subtype was obtained from the murine Hybridoma Cell Line RB96 3D6.32.2.4 (ATCC). Purified monoclonal 6E10, 4G8, and 4G8-HRP antibodies were obtained from Biolegend, San Diego. In preparation for antibody application to 5xFAD mice, 3D6 and K11 were recombinantly expressed with an IgG2a subtype in Freestyle 293-F cells (Thermo Fisher Scientific) by using the bicistronic vector pVITRO1-neo-mcs (InvivoGen). The isotype control antibody originally possesses an IgG2a subtype and was expressed in hybridoma cells. Antibody purifications from hybridoma or Freestyle 293-F supernatants have been done by Protein G affinity chromatography. Bound antibodies were eluted using 100 mM Glycine-HCl, pH 2.7, and dialyzed twice against PBS (138 mM NaCl, 8 mM Na_2_HPO_4_, 1.5 mM KH_2_PO_4_, 3 mM KCl, pH 7.1) overnight at 4 °C.

### Generation of anti-isoD7-Aβ antibody expressing hybridoma cells

We aimed at generating monoclonal antibodies, which bind to isoD7-Aβ, but not to D7-Aβ. For immunization, the peptide isoD7-Aβ (1–12)-Cys was used. The sulfhydryl group of terminal cysteine residue was used to conjugate the peptide to Bacterial Transglutaminase (BTG) as carrier. For generation of monoclonal antibodies, 8-week-old female BALB/c mice were immunized with the peptide-BTG-conjugates. Mice were immunized intraperitoneally with a water-in-oil emulsion that was prepared by emulsifying both antigens in equal volumes of Freund’s complete adjuvant (priming) or incomplete adjuvant (boosting). After mice showed sufficient antibody titer in serum, they were sacrificed by cervical dislocation. Spleens were aseptically removed, pooled, homogenized, and immortalized by cell fusion using myeloma cell line SP2/0-Agl4 purchased from the German Collection of Microorganisms and Cell Culture (DSMZ GmbH, Braunschweig). The resulting hybridoma clones were screened according their ability to bind isoD7-Aβ (1–18), but not the wild-type peptide Aβ (1–18). Screening of antigen binding occurred via direct enzyme-linked immunosorbent assay (ELISA) and surface plasmon resonance (SPR), immobilizing the antigen (~ 200 RU) onto a streptavidin sensor chip (GE Healthcare). Stable antibody-producing hybridomas have been selected and subsequently cloned for a second time by limited dilution in order to ensure the monoclonality of the hybridomas.

### Dot blot analysis

1.5 μl of Aβ peptides (100 ng/μl) were spotted on a nitrocellulose membrane and blocked for 1 h in blocking solution (5% (w/v) milk powder in TBST (TBS + 0.05% Tween 20 (v/v)). Antibodies 4G8, K11, 6E10, and 3D6 were diluted to 1 μg/ml in blocking solution and incubated with the membrane for 1 h, followed by 3 × 5 min washing steps with TBST. Anti-mouse antibody conjugated to alkaline phosphatase (AP) was added and incubated for 1 h, followed by 3 × 5 min washing steps and subsequent colorimetric detection of AP activity by addition of substrates BCIP (5-bromo-4-chloro-3-indolyl-phosphate) and NBT (nitro blue tetrazolium).

### Biophysical antibody characterization by SPR analysis

Binding kinetics (k_a_, k_d_, and K_D_) to different Aβ peptides were determined by using Biacore 3000 at a temperature of 25 °C. In order to capture the antibody of interest to a CM5 sensor Chip (GE Healthcare, Product code BR100012), approximately 15,000–20,000 RU of goat anti-mouse IgG (ThermoFisher Scientific, PA128555) were immobilized first. To immobilize the anti-mouse IgG, the carboxymethylated dextran surface of the sensor chip was activated by mixing 0.1 M *N*-hydroxysuccinimide (NHS) with 0.4 M *N*-ethyl-*N*′-(dimethylaminopropyl) carbodiimide hydrochloride (EDC) 1:1. EDC/NHS was applied to the sensor chip for 10 min with a flow rate of 10 μl/min. Goat anti-mouse IgG was diluted to 50 μg/ml in 10 mM sodium acetate, pH 5.5, and injected for 2 × 3 min contact time with a flow rate of 10 μl/min. After deactivation with 1 M ethanolamine, pH 8.5, for 2 × 7 min contact time with a flow rate of 10 μl/min, 0.1 M glycine, pH 1.7, was applied to the sensor chip with a flow rate of 30 μl/min for 3 min, followed by a washing step with HBS-EP buffer (GE Healthcare, Product code BR100188). Capturing of about 2000 RU anti-isoD7-Aβ antibodies occurred with a flow rate of 10 μl/min. To achieve this, antibodies were diluted to 25 μg/ml in HBS-EP buffer and applied to the sensor chip, followed by washing with HBS-EP until the RU signal remained constant. Binding to the clones K16, K23, K29, K119, K129, and K211 was determined by applying 1 nM to 1 μM of isoD7-Aβ (1–18) and 10 nM to 10 μM of Aβ (1–18), respectively, to the antibodies in a multi cycle kinetic analysis. The kinetic constants as well as the dissociation constant were calculated over all recorded sensorgrams using the 1:1 Langmuir binding model. The binding to antibody clone K11 was determined by applying 3–243 nM of isoD7-Aβ (1–18) and 10–810 nM of Aβ (1–18) in a single cycle kinetic analysis. The kinetic constants as well as the dissociation constant were calculated using the single cycle kinetic model. All evaluations were performed using the BIAevaluation 4.1.1 software.

#### Immunohistochemical analyses

##### Human brain tissue

The human brain tissue was provided by the former Brain Banking Centre Leipzig of the German Brain-Net, operated by the Paul Flechsig Institute of Brain Research, Medical Faculty, University of Leipzig. The procedure of case recruitment, the protocols, the informed consent forms, and autopsy have been approved (GZ 01GI9999-01GI0299). The diagnosis and staging of AD cases used in this study was based on the presence of neurofibrillary tangles [32] and neuritic plaques in the hippocampal formation and neocortical areas as outlined by the Consortium to establish a registry for AD (CERAD [33]) and met the criteria of the National Institute on Aging (NIA) on the likelihood of dementia [34]. For immunohistochemistry, temporal cortex (Brodmann area 22) from 6 AD cases (3 male, 3 female) and 4 aged-matched controls (2 male, 2 female) was used. Anatomical structures and cortical layers were identified using consecutive Nissl-stained sections.

##### Human brain tissue preparation

Tissue blocks of human temporal cortex were prepared in the frontal plane, fixed in 4% paraformaldehyde, and cryoprotected in 30% sucrose in 0.1 M phosphate buffer (pH 7.4) before sectioning. Thirty-micrometer-thick sections were cut on a freezing microtome and collected in 0.1 M phosphate buffer containing 0.1% sodium azide.

##### isoD7-Aβ or total-Aβ immunohistochemistry of human brain slices

All immunohistochemical procedures were performed on free-floating brain sections. Brain sections were pretreated with several rinses of PBS containing 0.05% Tween 20 (PBST). Then, sections were treated with 60% methanol, 2% H_2_O_2_ for 1 h prior to incubation with blocking solution (2% (v/v) BSA, 0.3% (w/v) milk powder, 0.5% (v/v) normal donkey serum). For analysis, the sections were incubated with 2 μg/ml of commercially available pan-Aβ antibody 6E10 (1:2000, Biolegend, San Diego) or with isoD7-Abeta specific antibody K11 in blocking solution overnight on a tumbler in a humid chamber at 4 °C. The following day sections were incubated with a biotinylated donkey anti-mouse secondary antibody (Dianova 1:1000) for 60 min at room temperature followed by the ABC method, which comprised incubation with ExtrAvidin-Peroxidase (Sigma-Aldrich, 1:2000) in PBST. Incubations were separated by washing steps (3 times, 5 min in PBST and 1 time, 5 min in Tris buffer (0.05 M, pH 8.0)). Binding of peroxidase was visualized by incubation with 2 mg 3,3′-diaminobenzidine (DAB), 20 mg nickel ammonium sulfate, and 2.5 μl 30%-H_2_O_2_ per 5 ml Tris buffer for 4 min resulting in black labeling at sites of Aβ deposition.

##### Murine brain tissue preparation

5xFAD mice were sacrificed by CO_2_ asphyxiation and transcardially perfused with phosphate-buffered saline (pH 7.4). After perfusion, brains were removed and post-fixed in 4% buffered paraformaldehyde for 48 h at 4 °C. After cryoprotection in 30% sucrose in 0.1 M phosphate buffer (pH 7.4) for 3 days coronal or sagittal sections from hippocampal region (30 μm) were cut on a cryomicrotome (Cryostar NX70) and collected in 0.1 M phosphate buffer containing 0.025% sodium azide.

##### isoD7-Aβ or total-Aβ immunohistochemistry of murine brain slices

Mouse brain slices were pre-treated with 60% methanol, 1% H_2_O_2_ (30 min), followed by washes in 0.1 M TBS (pH 7.4), and blocked in TBS containing 0.3% TritonX-100 and 5% normal goat serum for 30 min to reduce unspecific binding of antibodies. Incubation with primary antibody was performed in blocking buffer over night at 4 °C.

For staining of untreated 5xFAD mouse brain, slices were incubated with 2 μg/ml K11 or commercially available antibody 6E10 (1:500, Biolegend, San Diego), followed by application of biotinylated goat anti-mouse IgG (Thermo Fisher Scientific).

When analyzing 5xFAD brain slices, treated with K11_IgG2a, 3D6_IgG2a, or IgG2a isotype control, samples were incubated with 2 μg/ml isoD7-Aβ specific Ab K16 (isotype IgG1), 6E10 (isotype IgG1) or 0.5 μg/ml 3D6 (isotype IgG2b). Then slices were incubated with biotinylated anti-mouse IgG1 or anti-mouse IgG2b (1:1000 Dianova, Hamburg) in TBS with 2% (v/v) BSA for 1 h followed by incubation with ExtrAvidin-peroxidase (Sigma-Aldrich, 1:1000) in the same buffer. After 3 washing steps (5 min in TBS), immunostaining was performed by treatment of sections with 0.05% (w/v) of the chromogenic substrate 3,3′-diaminobenzidin (DAB) in 0.05 M Tris, pH 7.6, with 0.015% (v/v) H_2_O_2_ for 4 min.

##### Quantification of immunohistochemical labeling

The proportion of isoD7-Aβ and total-Aβ labeled structures (region of interest (ROI) in %) was quantified based on overall area of ROI by using the program BZ II Analyzer. All pictures were recorded by using the microscope Biorevo BZ-9000 (Keyence) with transmitted light modus and an exposure time of 1/200 s.

#### Passive immunization of 5xFAD mice

##### 5xFAD mouse model

5xFAD transgenic mice overexpress mutant human APP (695) with the Swedish (K670N, M671L), Florida (I716V), and London (V717I) FAD mutations along with human PS1 harboring two FAD mutations, M146L and L286V [35]. The mice are bred on a C57Bl/6J background and only heterozygous 5xFAD mice were used, with wild-type littermates as controls. Mouse genotypes were checked twice, before and after the experiments. Mice were group-housed in individually ventilated cages, with ad libitum access to water and food. Mice were kept on a regular 12/12 h light-dark cycle and behavioral experiments were performed during the light phase.

Two trials were conducted in gender- and age-matched 5xFAD mice. For immunization, recombinant expressed antibodies with an IgG2a isotype were used. In an initial short treatment trial, 500 μg, 150 μg K11_IgG2a, and 500 μg IgG2a isotype control were applied intraperitoneally once a week to 3-month-old females. Mice were sacrificed 1 week after 12 weeks treatment. In the second, long treatment trial, 3-month-old female 5xFAD mice were treated for 38 weeks with 12 mg/kg (~ 300 μg) K11_IgG2a, 3D6_IgG2a, and IgG2a isotype control. In addition to the read out by ELISA, different behavioral tests have been performed. Therefore, wild-type mice have also been treated with 12 mg/kg isotype control per week, in order to preclude any influence of the isotype control antibody, handling, and injections.

##### Sample collection

Mice were sacrificed using CO_2_ inhalation 1 week after the final immunization. Whole blood was collected in lithium-heparin tubes and centrifuged for 10 min at 1920×*g* and 4 °C. Plasma was then collected and snap frozen. After perfusion with PBS, mouse brain was removed and divided sagittally. The right hemisphere was treated with formaldehyde, cryopreserved, and used for immunohistochemical staining. The left one was snap frozen and prepared for ELISA analysis. Cerebellum was also snap frozen and homogenized in ELISA Blocker.

#### Behavioral tests

##### Elevated plus maze (EPM)

Test animals were placed with their head to the end of a defined closed arm of an elevated, plus-shaped (+) maze with two open and two enclosed arms (Biobserve GmbH, Bonn, Germany). Bedding was applied to the maze, and it was changed for each tested mouse. Spontaneous exploration of the maze was recorded. The time the animals spent in the open arms was summed up in order to calculate the percentage of time spent in exposed area. A movement was defined as arm entry when the entire animal (except tail) entered the open arm.

##### Fear conditioning

This test is designed for the measurement of learning and memory in which an aversive stimulus (electrical shock) is associated with a particular neutral stimulus (tone) addressing hippocampal and extra-hippocampal learning. Successful learning leads to the evocation of state of fear (freezing) by the neutral stimulus alone (memory of cue) or even by exposition to the same environment where the aversive stimulus occurred (contextual memory). Test animals were placed in an automated Fear Conditioning System (TSE Systems, Bad Homburg, Germany). The training phase consisted of habituation time of 210 s followed by a continuous sound (100 dB, 28 s) and an electric foot shock (0.7 mA for 2 s). After 24 h, test animals were again placed in the Fear Conditioning System and the contextual memory evident as freezing was recorded. One hour later, animals were put back into the system with a changed context (black walls, no grid on the floor). After free exploration for 180 s, tested mice were subjected to the adverse sound as presented on the training day but without foot shock. The memory of cue was recorded as freezing time. Thorough cleaning of all components between individual mice was performed to avoid distraction from previously tested mice.

##### Pole test

The pole test was used to investigate motor coordination. The 50-cm high pole (diameter 1.5 cm) was placed in a cage whose ground is completely covered with bedding. The mice were first placed for 20 s on the ground of the cage for short exploration. Afterwards, the mice were placed on the tip of the pole with their head directed to the top. Immediately after unhand, time was counted until animals turned around (defined as every single paw is directed to the ground). In addition, the down time was also recorded (defined as all paws have contact to the ground). The maximum time for the test is 120 s. Falling, springing, and other deviating behavior was counted as 120 s. Each animal was tested 5 times in a row. After testing, all mice were returned to their home cage, the pole was cleaned, and bedding was changed. The mean of turn time and down time were calculated.

##### Morris water maze (MWM) test

Test animals were placed in a circular pool (TSE Systems, Bad Homburg, Germany) filled with water (26 ± 0.5 °C). The mice are required to find an invisible platform in the target quadrant that allows them to escape the water. Therefore, the animals use distal cues on the edge of the pool as points of reference to locate themselves. The circular pool is divided into 4 equal quadrants (target quadrant and 3 test quadrants), which can be visually distinguished by the cues. Test animals were placed into the first quadrant and the time was counted until they reached the platform. In case they did not reach the platform after 60 s, the mice were led to it. After at least 5 min break with drying under red light, test animals were placed into the second quadrant and exposed to the same procedure. The animals were allowed to pause again, followed by putting them in quadrant 3, followed by another pause and putting them again in quadrant 2. Each day the mice performed 4 swim tests (1 × quadrant 1, 2 × quadrant 2, 1 × quadrant 3). Swim tests were done on 4 consecutive days. Individual swim time and speed are recorded by a video tracking system (Biobserve, Bonn Germany). The mean time to reach the platform for each mouse and day was calculated and graphically illustrated as learning curve.

#### ELISAs

##### Generation of Aβ (40) and Aβ (42) fibrils and preparation for ELISA analysis

Aβ (1–42) and Aβ (1–40) peptides were dissolved in 1,1,1,3,3,3-hexafluoro-2-isopropanol (HFIP). The HFIP was evaporated under a fume hood overnight. Peptide pellets were dissolved to a final concentration of 10 μM as already described in Piechotta et al., 2017. Fibril formation was confirmed by transmission electron microscopy. For ELISA analysis, fibrils were resolved by addition of 19 volumes 70% formic acid and incubated for 10 min at room temperature, followed by neutralization with 130 volumes 3.5 M Tris.

##### isoD7-Aβ and total Aβ-specific ELISA

In order to prepare mouse brain for isoD7-Aβ and total Aβ-ELISA analysis, the left hemisphere was homogenized in T-Per buffer (Tissue Protein Extraction Reagent, Thermo Fisher Scientific) at a concentration of 50 mg/ml with Protease Inhibitor Cocktail Tablets (Roche) by using a Precellys homogenizer (VWR), followed by sonification for 10 s. The homogenate was centrifuged for 1 h at 100,000×*g*, thereby yielding the T-Per fractions. The resulting pellet was dissolved to 150 mg/ml in 5 M guanidine hydrochloride (5 M GdmCl), followed by an incubation step in an overhead shaker for 3 h at room temperature. After a next centrifugation step (1 h at 100,000×*g*), supernatant (5 M GdmCl fractions) was collected and stored at − 20 °C until use.

Coating antibodies K11 or 3D6 respectively were diluted in PBS to 2 μg/ml and immobilized on polystyrene 96-well microtiter plates (Nunc Maxisorp, flat-bottom) overnight at 4 °C. Blocking occurred for 2 h at 4 °C with ELISA Blocker (Thermo Fisher Scientific). Samples were diluted in ELISA Blocker + Tween 20 (Thermo Fisher Scientific). For preparation of the standard curve, synthetic isoD7-Aβ (1–30) was serially diluted from 150 pg/ml down to 1.6 pg/ml and added to the wells in duplicate. After an incubation period of 2 h at 4 °C, plates were washed six times with TBST. For detection of bound Aβ species, the HRP-conjugated anti-Aβ-antibody clone 4G8 (Biolegend, San Diego) was diluted to a final concentration of 1 μg/ml in ELISA Blocker + Tween 20 and incubated for 1 h at 4 °C with the samples. After six washing steps with TBST, a color reaction with commercially available HRP substrate TMB (SureBlue Reserve TMB Microwell Peroxidase Substrate (1-component), KPL) was performed and stopped by the addition of 1.2 N H_2_SO_4_. Absorption at 450/540 nm was determined by a Tecan Sunrise plate reader. The standard curve was calculated from measured absorption by a 4-Parameter-Logistic-Fit: *y* = A2 + (A1 − A2)/(1 + (*x*/*x*0)^*p*). When analyzing the isoD7-Aβ and total Aβ amounts in biological samples, sample dilutions resulting in adsorption signals within the linear range of the standard curve were selected.

##### Aβ(*X*–42)-specific ELISA

Aβ(*X*–42) ELISA was performed as described above (isoD7-Aβ and total Aβ-specific ELISAs) with the following exceptions: Antibody 4G8 (Biolegend) was used as Coating antibody and 12F4-Biotin (Biolegend) as detecting antibody in a concentration of 1 μg/ml. Detecting antibody 12F4-Biotin was pre-incubated with 2 μg/ml Streptavidin-HRP (Sigma-Aldrich) for 15 min at room temperature before application to the samples. For preparation of standard curve, synthetic Aβ(*X*–42) was diluted from 1 μg/ml to 1.37 pg/ml.

##### K11_IgG2a and 3D6_IgG2a concentration measurement in cerebellum

The determination of antibody concentrations in brain samples was performed as already described [36]. Since antibody 3D6 does not discriminate between isoD7-Aβ and D7-Aβ, we used 20 ng biotinylated isoD7-Aβ (1–18) for coating.

## Results

### Antibody generation and initial characterization

**Fig. 1:**
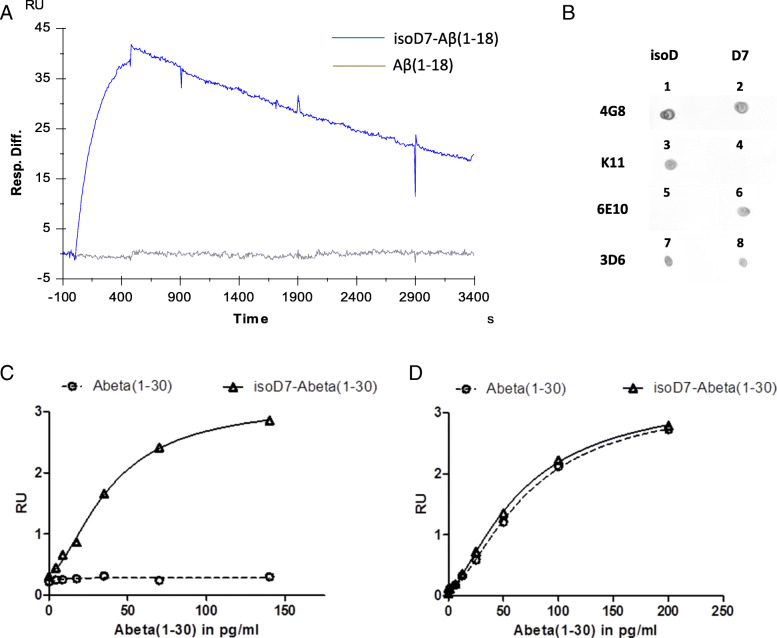
Antibody characterization and ELISA development. **a** The interaction of Aβ peptides with K11 was analyzed at a Biacore 3000 at 25 °C. Goat anti mouse IgG was immobilized on a CM5 sensor chip, followed by binding of mouse anti-isoD7-Aβ antibody K11. The binding of isoD7-Aβ (1–18) and Aβ (1–18) to K11 was determined by separate injections of 100 nM of each Aβ peptide. **b** Analysis of different antibodies for specificity in Sandwich ELISAs. 150 ng of Aβ peptides (1—isoD7-Aβ (1–40); 2—Aβ (1–40); 3, 5, 7—isoD7-Aβ (1–30); 4, 6, 8—Aβ (1–30)) was spotted on a nitrocellulose membrane which was blocked in blocking solution for 1 h. Antibodies 4G8, K11, 6E10, and 3D6 were diluted to 1 μg/ml in blocking solution and incubated with the membrane for 1 h. Anti-mouse antibody conjugated to AP was added and incubated for 1 h, followed by 3 × 5 min washing steps and subsequent colorimetric detection of AP activity by addition of substrates BCIP and NBT. **c**, **d** Establishment of Sandwich ELISAs for quantification of isoD7-Aβ and total Aβ concentrations. K11 (**c**) and total Aβ specific antibody 3D6 (**d**) were diluted to 2 μg/ml and immobilized on microtiter plates overnight at 4 °C. Blocking occurred for 2 h at room temperature. Standard peptides isoD7-Aβ (1–30) and Aβ (1–30) were serially diluted from 150 pg/ml down to 1.6 pg/ml. After an incubation period of 2 h at 4 °C, plates were washed six times with TBS-T. HRP-conjugated anti-Aβ antibody clone 4G8 was added in a final concentration of 1 μg/ml and incubated for 1 h at 4 °C. After washing with TBS-T, a color reaction with TMB was performed and stopped by the addition of 1.2 N H_2_SO_4_. The standard curve was calculated from measured absorption at 450/540 nm by a 4-Parameter-Logistic-Fit: *y* = (A2 + (A1 − A2)/(1 + (*x*/*x*0)^*p*)

For generation of cell lines producing anti-isoD7-Aβ antibodies, we immunized 8-week-old female BALB/c mice with isoD7-Aβ (1–12)-Cys, conjugated to BTG as carrier. Spleens were aseptically removed from animals possessing the highest titer, homogenized, and immortalized by cell fusion with the myeloma cell line SP2/0-Agl4. The resulting hybridoma clones were screened according their ability to bind isoD7-Aβ (1–18), but not the wild-type peptide Aβ (1–18). Different anti-isoD7-Aβ antibody producing hybridoma cell lines were obtained. Kinetic characterization (see Additional file 1 for summary of antibodies, subtypes, kinetic data and specificity) and immunohistochemical staining of human AD brain samples (see Additional file 2 for the respective images) as well as transgenic mouse tissue led to the identification of the preferred candidate molecule K11. This antibody selectively binds isoD7-Aβ (1–18) in comparison to the non-modified form (Fig. 1a–c). In order to get a detailed overview of antibody specificity and possible cross reactivity, we determined kinetic data for the binding of different Aβ peptides with varying lengths and amino acid modifications (Table 1). One sensorgram, showing the binding of K11 to different concentrations of isoD7-Aβ (1–18), is shown exemplarily in Additional file 3. While isoD7-Aβ is bound with high affinity, binding of its D-stereoisomer isod7-Aβ and non-modified Aβ occurs with an about 450-fold increased *K*_D_ value. Furthermore, binding of the epitope isoD7-Aβ (1–12) is independent from the C-terminal peptide length. The antibody K11 displays a 25-fold lower affinity to the murine Aβ variant, which displays different amino acid residues at positions 5, 10, and 13. Other prominent post-translational modifications of the Aβ peptide, which are close to amino acid 7 and also found in AD patients as well as in mouse models, are nitrated tyrosine at position 10 (3NY10-Aβ) [37], phosphoserine at position 8 (phosphoSer8-Aβ) [38], and pyroglutamate at position 3 (pGlu3-Aβ) [39, 40]. Surprisingly, stronger binding occurs if a pGlu-modification at position 3 of the Aβ-peptide is present. On the other hand, binding is prevented by the presence of phosphoSer8 and considerably impaired by the presence of 3NY10.

**Table 1:** Kinetic analysis of K11 binding to different Aβ peptide variants

Peptide	***K***_**D**_ value [nM]
isoD7-Aβ(1–18)	6
N7-Aβ(1–18)	2560
Aβ(1–18)	2700
isoD7-Aβ(1–40)	4
Aβ(1–40)	1670
isod7-Aβ(1–17)	2690
mouse isoD7-Aβ(1–18)	153
isoD7-3NY10-Aβ(1–18)	652
isoD7-PhosphoSer8-Aβ(1–18)	No binding
pGlu3-isoD7-Aβ(3–18)	2
pGlu3-Aβ(3–18)	402

Binding affinities of immobilized antibody K11 to different Aβ peptides were determined by using a Biacore 3000 at 25 °C. Goat anti mouse IgG was immobilized on a CM5 sensor chip, followed by binding of mouse anti isoD7-Aβ antibody K11. Kinetic constants were determined by applying Aβ peptides at different concentrations and calculated from the combined set of data by using BIAevaluation software (Biacore AB)

*isoD7* l-isoaspartate, *isod7* d-isoaspartate, *3NY10* nitrotyrosin at position 10, *PhosphoSer* phosphoserine at position 8, *pGlu3* pyroglutamate at position 3

### Quantification of isoD7-Aβ and total Aβ by ELISA

In order to establish an assay for the quantification of Aβ plaque load on the one hand and for determination of the percentage of isoD7-Aβ content in biological samples on the other hand, we developed ELISAs for isoD7-Aβ and total Aβ quantification. First, we evaluated the reactivity of several commercially available antibodies with isoD7-Aβ and non-modified Aβ by dot blot analysis. The monoclonal antibody 3D6 is specific for the intact N-terminus of the Aβ peptide; 4G8 recognizes amino acids 17–24. Both antibodies bind Aβ independently from amino acid 7 (Fig. 1b, d). The antibody 6E10 was raised against Aβ (1–16) and is specific to non-modified D7-Aβ (Fig. 1b).

We used the antibodies 3D6 and K11 in combination with 4G8 to establish ELISAs (Fig. 1c, d). By using the anti-isoD7-Aβ specific antibody K11 for coating, we developed an indirect Sandwich ELISA for the quantitative detection of isoD7-Aβ down to 1.6 pg/ml. Figure 1c shows a characteristic standard curve for the isoD7-Aβ specific ELISA. The graph further shows that non-modified Aβ is not detected. The additional development of a total Aβ ELISA, which detects Aβ independently from the isoD7 modification, allows the calculation of the percentage of isoD7-content in biological samples (Fig. 1d).

### Formation of isoD7-Aβ within Aβ (1–40/42) fibrils

To address the question whether isomerization of aspartate 7 can occur within the fibrillary structure of the Aβ peptide, we generated Aβ (1–40) as well as Aβ (1–42) fibrils and incubated them in PBS at 37° for 1 year. Successful formation of Aβ-fibrils was evidenced by transmission electron microscopy (Additional file 4). During the incubation period samples were taken, dissolved to monomers by formic acid treatment and subsequently analyzed concerning the respective percentage of isoD7-Aβ content by using the newly developed isoD7-Aβ and total Aβ ELISAs. Additional file 5 depicts the constant spontaneous formation of isoD7-Aβ within the fibril structure of Aβ (1–40) as well as Aβ (1–42).

### Increased isoD7-Aβ formation in D678N_APP

Members of the Japanese-Tottori FAD pedigree carry the missense mutation D678N-APP which results in the production of N7-Aβ [28]. In order to investigate if the expression of N7-Aβ leads to an enhanced formation of isoD7-Aβ, we transfected HEK293 cells with D678N_APP, followed by the quantitative determination of total Aβ and isoD7-Aβ in the cell culture supernatant. By evaluating the isoD7-Aβ content in the supernatants, we show that transfection of D678N_APP leads to a strong increase in isoD7-Aβ formation compared to wild-type APP transfected cells (Additional file 6). Moreover, a massive increase in isoD7-Aβ formation was observed within 24-h incubation time from day 4 to day 5 after transfection. Hence, given the specific pathogenicity of this Aβ-species, increased formation of isoD7-Aβ might be the underlying reason for the disease propagating properties of D678N-APP in the Japanese-Tottori FAD pedigree.

### isoD7-Aβ is present in brain of human AD patients

**Fig. 2:**
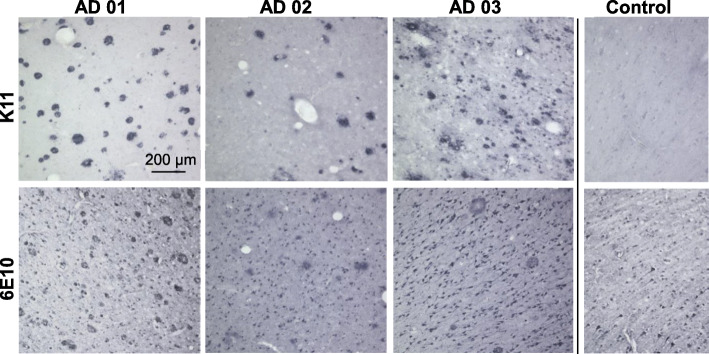
Immunohistochemical analysis of Aβ deposits in human brain samples. Brain slices of different AD patients and an age matched healthy control, respectively, were incubated with the antibodies 6E10 or K11, followed by application of biotinylated anti-mouse IgG

The presence of isoD7-Aβ in brains of human AD patients was already shown before [12, 13]. However, due to the high density of Aβ peptides in AD brain, even a low antibody cross reactivity will result in the detection of other Aβ peptide species, e.g., in amyloid plaques. Therefore, it was of essential importance to employ a highly specific monoclonal antibody to demonstrate the presence of isoD7-Aβ in AD brain. We performed immunohistochemical staining of a variety of human brain tissue samples from several AD cases in order to test specific binding and to choose the lead candidate out of several anti isoD7-Aβ antibodies based on specific signal to background labeling ratio (Additional file 2). Figure 2 shows exemplarily the immunohistochemical staining of brain samples from 3 different AD cases with K11 in comparison to 6E10. The latter showed a more widespread neuronal staining besides binding of amyloid plaques (Fig. 2). In contrast, K11 exclusively stains amyloid plaques in human brain samples (Fig. 2).

### isoD7-Aβ is present in 5xFAD mouse brain and increases with aging

**Fig. 3:**
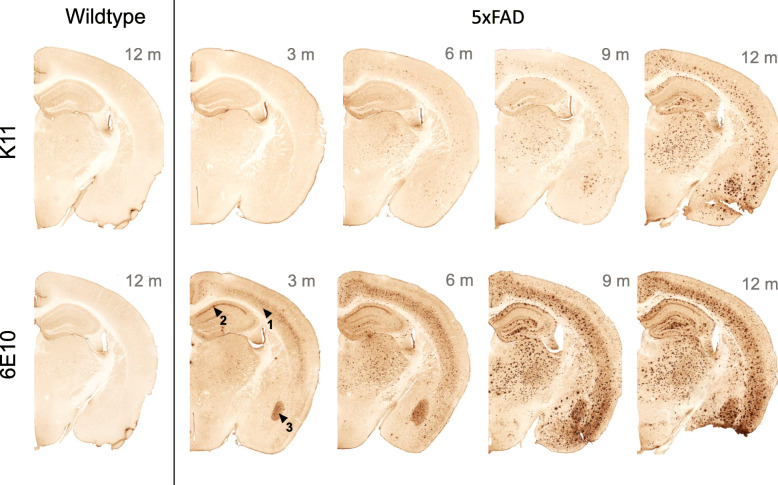
Immunohistochemical analysis of Aβ deposition in brain samples from 5xFAD and wild-type mice. Brain slices of wild type or 5xFAD mice were incubated with the antibodies 6E10 and K11, respectively, followed by application of biotinylated anti-mouse IgG

In order to demonstrate the presence of isoD7-Aβ in a mouse model possessing amyloid pathology, sections of brain tissue from 5xFAD mice from different ages were analyzed. Figure 3 shows immunohistochemical staining using the anti-isoD7-Aβ antibody K11 and the non-modified Aβ-specific antibody 6E10. First amyloid deposits are observed by 6E10 staining approx. 3 months after birth in 5xFAD mice (Fig. 3). IsoD7-Aβ containing plaques were initially observed in 6-month-old animals. In contrast to the isoD7-Aβ-specific antibody, 6E10 also reacts with cells in cortex (Fig. 3, arrow 1), hippocampus (Fig. 3, arrow 2), and basolateral amygdala (Fig. 3, arrow 3) most likely due to recognition of human APP expression in these areas. K11 exclusively stains extracellular plaques consisting of deposited and aged Aβ species. Quantity and expansion of amyloid plaques further proceeded during aging of 5xFAD mice, shown by 6E10- and K11-positive staining (Fig. 3). There was no staining in wild-type mouse brain by K11 and 6E10.

### Application of K11_IgG2a in a 5xFAD mouse model—pilot study

**Fig. 4:**
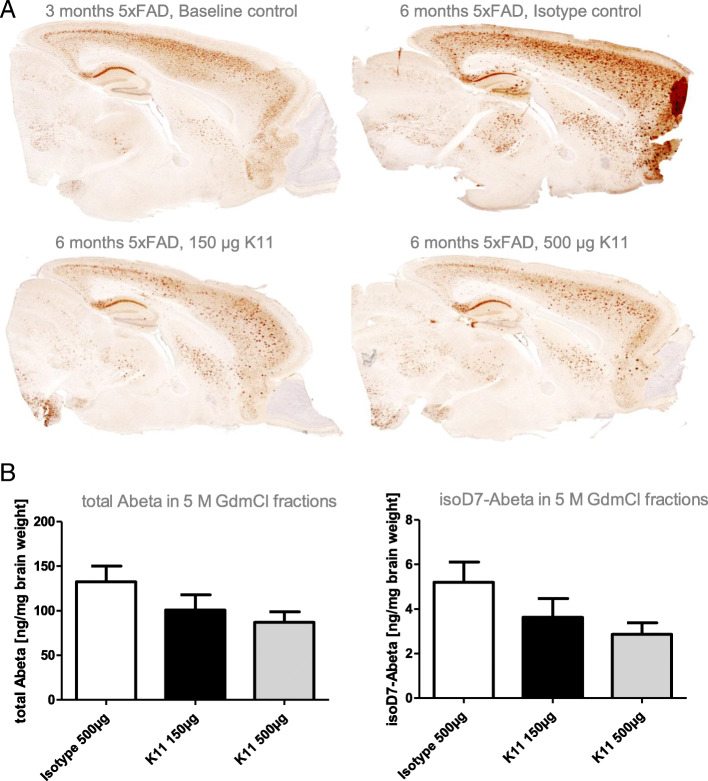
Analysis of Aβ deposits in brain samples from 5xFAD mice treated with K11_IgG2a and isotype control. Three-month-old 5xFAD mice were treated intraperitoneally once a week with 500 μg, 150 μg K11_IgG2a, or 500 μg isotype control. After 12 weeks of treatment, mice were sacrificed. **a** Immunohistochemical analysis—the right hemisphere was treated with paraformaldehyde and slices were prepared. Brain slices have been incubated with antibody 6E10, followed by application of biotinylated anti mouse IgG1. **b** Quantification of total Aβ and isoD7-Aβ peptides—the left hemisphere was homogenized in T-Per buffer, followed by centrifugation. The pellet was resuspended in 5 M GdmCl, again centrifuged and the supernatants applied to a total Aβ (left) and isoD7-Aβ (right) specific ELISA. Sample size was at least 6 animals per group. The error bars represent SEM

In the 5xFAD mouse model, isoD7-Aβ containing plaques appear between 3 and 6 months of age (Fig. 3). In order to evaluate the capability of K11 in reducing Aβ plaque load, we applied the antibody in two different doses (150 μg and 500 μg / mouse) to 3-month-old female 5xFAD mice once a week for a total of 12 weeks. Due to the fact that IgG2a antibody isotypes possess the highest plaque reducing activity in passive immunotherapy in AD mouse models [41, 42], we recombinantly expressed K11 as IgG2a isotype. Mice were sacrificed 12 weeks after treatment; the brains were removed and divided sagittally. One hemisphere was used for immunostaining and one hemisphere was homogenized for subsequent ELISA analysis, resulting in T-Per and 5 M GdmCl fractions. The effectiveness of lowering isoD7-Aβ and total Aβ level was also investigated by histological end points (Fig. 4a). As shown by dot blot analysis in Fig. 1b, the commercially available antibody 6E10 is specific for unmodified Aβ peptides and does not react with isoD7-Aβ. Figure 4a shows immunohistochemical staining of brain slices from K11_IgG2a-treated 5xFAD mice by using 6E10. Despite the fact that 6E10 is not recognizing isoD7-Aβ, a clear reduction of Aβ plaque load was shown after K11_IgG2a treatment (Fig. 4a). This clearly demonstrates that targeting isoD7-Aβ results in a simultaneous reduction of non-modified total Aβ.

T-Per contains a mild detergent and was shown to extract target proteins from various cellular compartments, for example from plasma membrane. Mainly monomeric and oligomeric Aβ peptides are supposed to be present in the T-Per fraction. GdmCl is a strong denaturant of folded protein structures, mainly Aβ peptides from fibrillary structures which are predicted to dissolve in 5 M GdmCl. By using isoD7- and total Aβ-specific ELISAs, a clear dose-dependent reduction of isoD7-Aβ was observed after immunization. Interestingly, also total Aβ plaque load was reduced when compared to the isotype control group (Fig. 4b). In 5 M GdmCl fractions, isoD7-Aβ content is reduced to 55.1% at the highest antibody concentration and to 69.8% at the lower dose (Fig. 4b). Total Aβ levels are also reduced to 65.6% and 76.1%, respectively (Fig. 4b). Same trends were seen in T-Per fractions, isoD7-Aβ is reduced to 62.1% at high dose and 70.8% at low dose; total Aβ is lowered to 71.0% and 74.0%, respectively (see Additional file 7).

### Application of K11_IgG2a in a 5xFAD mouse model—long-term treatment

In a second treatment trial, 3-month-old 5xFAD mice were subjected to 38 weeks of weekly intraperitoneal injections of 12 mg/kg K11_IgG2a. As positive control 12 mg/kg per week 3D6_IgG2a, the parent molecule of bapineuzumab, was used. In addition, a control group of 5xFAD received weekly injections of an IgG2a isotype antibody. In contrast to 6-month-old 5xFAD mice, 11–12-month-old animals show considerable memory deficits in comparison to wild-type animals. Therefore, long-term treatment provides the opportunity for additional behavioral testing besides biochemical and histopathological read-outs. In order to determine cognitive function in wild-type mice and to preclude any unexpected influence of antibody applications, an additional group of wild-type mice was subjected to the weekly treatment with 12 mg/kg isotype control.

**Fig. 5:**
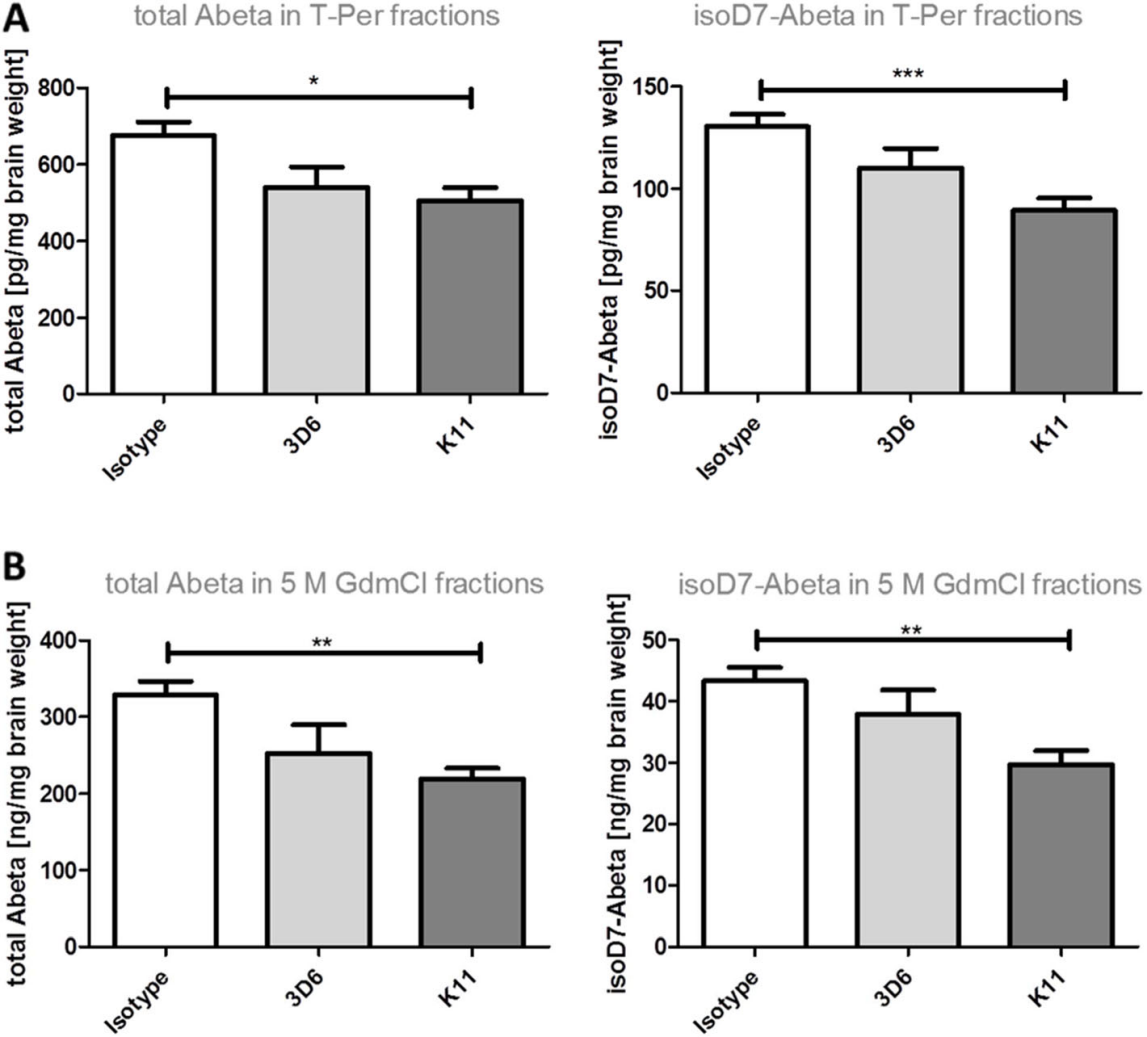
Quantification of total Aβ and isoD7-Aβ peptides in 5xFAD mice brain treated with K11_IgG2a, 3D6_IgG2a, and isotype control. Three-month-old 5xFAD mice were treated weekly by intraperitoneal injections with 12 mg/kg K11_IgG2a, 3D6_IgG2a, and isotype control. After 38 weeks of treatment, mice were sacrificed and the left hemisphere was homogenized in T-Per buffer, followed by centrifugation. **a** In the resulting supernatants (T-Per fractions), the amounts of total Aβ and isoD7-Aβ were analyzed by ELISA. **b** The pellet was dissolved in 5 M GdmCl, again centrifuged, and the supernatants applied to a total Aβ- or isoD7-Aβ-specific ELISA. For statistical analysis Tukey’s multiple comparison test was used. Sample size was at least 10 animals per group. *p ≤ 0.05; **p ≤ 0.01; ***p ≤ 0.001. The error bars represent SEM

ELISA analysis of soluble T-Per fractions shows a significant reduction of isoD7-Aβ to 68.4% in K11_IgG2a-treated animals in comparison to the isotype control group. In T-Per fractions, total Aβ levels are also significantly reduced to 74.8% by K11_IgG2a treatment (Fig. 5a). ELISA analysis of 5 M GdmCl brain tissue fractions of 5xFAD mice treated with our anti-isoD7-Aβ antibody K11_IgG2a showed that total Aβ levels (lowered to 66.8%) as well as isoD7-Aβ levels (lowered to 68.6%) are significantly reduced in the left hemisphere in comparison to the isotype control group (Fig. 5b). Treatment with the positive control antibody 3D6_IgG2a also resulted in lower Aβ contents in T-Per as well as 5 M GdmCl fractions, but no significant reduction was observed.

**Fig. 6:**
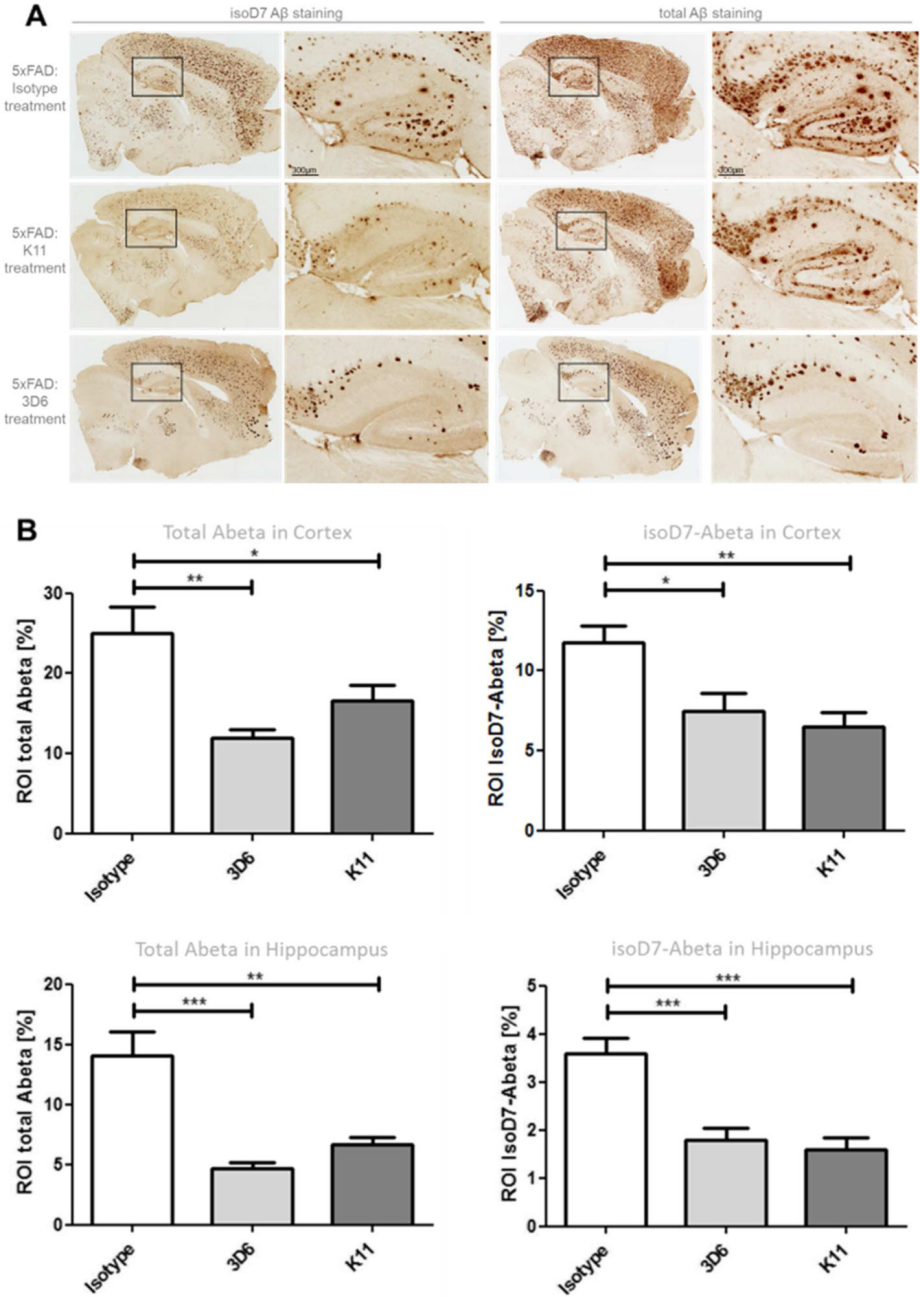
Immunohistochemical analysis of Aβ aggregates in hippocampal brain sections from 5xFAD mice treated with K11_IgG2a, 3D6_IgG2a, and isotype control. **a** Immunohistochemical analysis. Three-month-old 5xFAD mice were treated intraperitoneally once a week with 12 mg/kg K11_IgG2a, 3D6_IgG2a, or isotype control. After 38 weeks of treatment, mice were sacrificed. The right hemisphere was treated with paraformaldehyde, cryoconserved, and sliced. ROI in hippocampal brain slices were selected by staining with anti-isoD7-Aβ antibody K16 or total Aβ antibody 3D6 as indicated, followed by application of biotinylated anti-mouse IgG2b or anti-mouse IgG1, respectively. **b** Quantitative evaluation. Area of isoD7- or total Aβ containing peptides (ROI in %) was quantified based on overall area of ROI by using the program BZ II Analyzer. For statistical analysis, Tukey’s multiple comparison test was used. Sample size was 3 slices per brain and at least 10 animals per group. *p ≤ 0.05; **p ≤ 0.01; ***p ≤ 0.001. The error bars represent SEM

Because ELISA data represent the Aβ content in the whole hemisphere, we additionally performed immunohistochemical staining of brain slices in order to specifically localize sites of antibody action (Fig. 6a). Similar to the obtained ELISA data, immunohistochemical staining showed a significant reduction of Aβ plaque load in cortex and hippocampus after K11_IgG2a as well as 3D6_IgG2a treatment (Fig. 6b). The most prominent immunization effects were detected in the hippocampus which is also particularly affected in AD. Hippocampal staining with anti-isoD7-Aβ antibody revealed the highest reduction of isoD7-Aβ containing plaques from 3.6% surface area (isotype control group) to 1.6% surface area for the K11_IgG2a-treated group and to 1.8% surface area for the 3D6_IgG2a-treated animals (Fig. 6b). Interestingly, immunohistochemical staining using total Aβ-detecting antibody 3D6 demonstrated an even higher reduction of total Aβ in hippocampal and cortical ROI after 3D6_IgG2a treatment in comparison to K11_IgG2a treatment. Furthermore, the plaque morphology of 3D6_IgG2a-treated animals was different, with a more centralized and compact appearance, whereas the plaques in K11_IgG2a-treated mice were more diffuse and expanded (Fig. 6a). This is consistent with the observations of De Mattos et al., who described that 3D6_IgG2b failed to bind to already existing plaques because it becomes saturated by the soluble Aβ forms surrounding the plaque like a dense cloud [43]. Our isoD7-Aβ-specific antibody very likely binds within the plaque core and the plaque surrounding Aβ seems less affected.

Together, ELISA and immunohistochemical analysis revealed a significant reduction of Aβ load by K11_IgG2a and 3D6_IgG2a antibody treatment. In order to investigate the influence of reduced Aβ levels on cognitive function, a number of behavioral tests were performed.

**Fig. 7:**
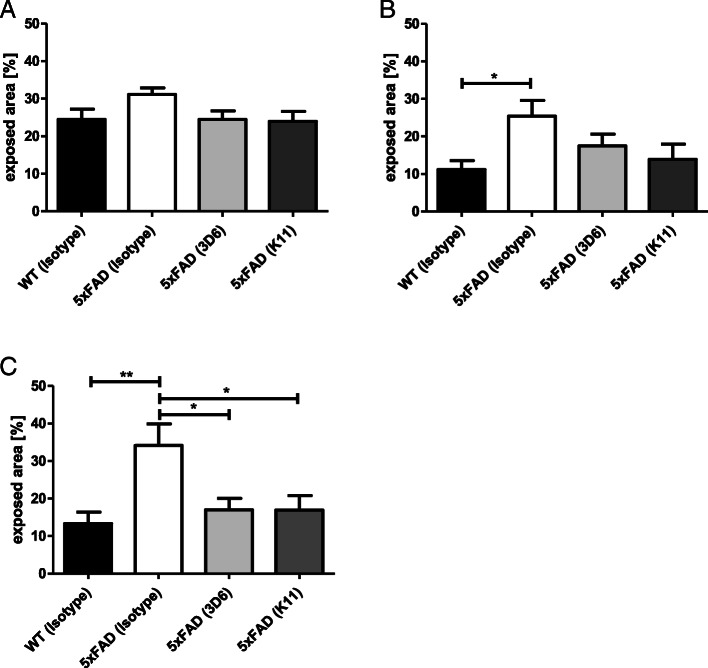
Elevated plus maze (EPM) test of mice treated weekly with 12 mg/kg K11_IgG2a, 3D6_IgG2a, and isotype control, respectively. This test was performed 3 times with each animal, at the age of 6 (**a**), 9 (**b**), and 12 months (**c**). Antibody-treated 5xFAD groups were compared with wild-type animals treated with 12 mg/kg isotype control. Test animals were placed with their head to the end of a defined closed arm of an elevated, plus-shaped (+) maze with two open and two enclosed arms. During the next 10 min, every movement of test animals was recorded by a video tracking system. The time the animals spent in the open arms was summed up in order to calculate % in exposed area. For statistical analysis, Tukey’s multiple comparison test was used. Sample size was at least 9 animals per group. *p ≤ 0.05; **p ≤ 0.01. The error bars represent SEM

The EPM test is a tool for the measurement of anxiety. Since this test is based on the animal’s aversion to open spaces and not on anxiety responses that rely upon the presentation of noxious stimuli, there is no learning effect on test animals and this test can be performed repeatedly. A difference in anxiety behavior between 5xFAD and wild-type mice is first detectable at the age of 6 months (Fig. 7a). In 9-month-old animals, we determined significant differences between these groups (Fig. 7b). At the age of 12 months, treatment with K11_IgG2a (16.9% of time in exposed area) as well as 3D6_IgG2a (17.0% of time in exposed area) reduced the time the animals spent in open arms to similar levels as the wild-type mice (13.3% of time in exposed area). In contrast, isotype control-treated mice spent 34.1% of time in exposed area (Fig. 7c). Importantly, the entire number of arm entries of each 5xFAD group, which corresponds to the mobility of mice, showed no difference between isotype-, K11_IgG2a-, and 3D6_IgG2a-treated mice, substantiating the finding that application of 3D6_IgG2a and K11_IgG2a altered the behavioral phenotype of 5xFAD (Additional file 8).

It is generally accepted that contextual fear conditioning (CFC) depends on hippocampal function [44]. Since the hippocampus is particularly affected in AD as well as in the 5xFAD mouse model, we decided to prove the effect of passive immunotherapy on cognitive deficits by means of CFC. 5xFAD animals treated with isotype control show significantly shorter freezing times after subjection to the neutral stimulus (15%) in comparison to the wild-type group (38%) (Additional file 9). Treatment with K11_IgG2a enhances freezing times to 32%; treatment with 3D6_IgG2a had lower effects (26%). Both treatment groups are statistically not distinguishable from wild-type mice (Additional file 9A). Considering the memory of cue, there was no significant difference in the freezing time even between the wild-type and isotype control groups (Additional file 9B). Hence, the memory of cue-test is not suitable for analyzing cognitive deficits in 11-month-old 5xFAD mice.

**Fig. 8:**
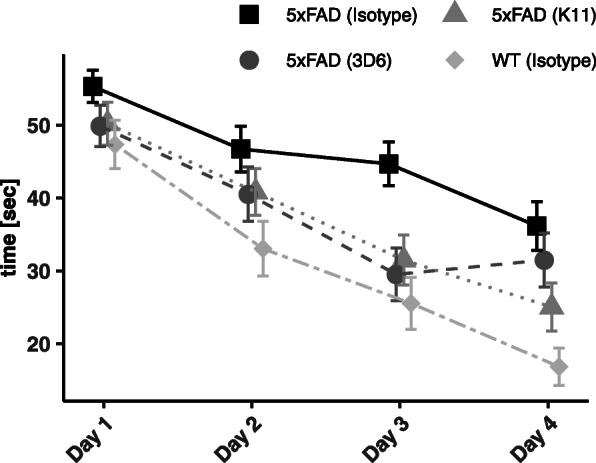
Morris water maze test of mice treated weekly for 38 weeks with 12 mg/kg K11_IgG2a, 3D6_IgG2a, and isotype control. Learning curves were obtained by delineation of the average trial time for 4 trials per day at days 1–4. Antibody-treated 5xFAD groups are compared to wild-type animals treated with 12 mg/kg isotype control. Test animals were placed in a circular pool and are required to find an invisible platform that allows them to escape the water. The circular pool is divided into 4 equal quadrants. Test animals were placed into the first quadrant and time was counted until they reached the platform. After at least 5-min pause, test animals were placed into the second quadrant and exposed to the same procedure. The animals were allowed to pause again, followed by putting them in quadrant 3, followed by another pause and putting them again in quadrant 2. At the end, time until the test animals reached the platform was analyzed for every mouse in 4 trials per day. The error bars represent SEM

In order to analyze spatial learning and memory of antibody-treated 5xFAD mice, we performed the Morris water maze (MWM). Mice were placed in a circular pool and were required to find an invisible platform that allowed them to escape the water. The daily performance provides hippocampus-dependent learning, including the acquisition of spatial memory. Figure 8 shows the average trial time until animals reached the platform on days 1–4, after exposing them to an everyday procedure consisting of 4 trials per day. The resulting learning curves represent the speed of task acquisition. Wild-type animals showed a steep curve which represents faster task acquisition, whereas isotype-treated 5xFAD animals revealed a shallower curve which represents a deficit in spatial learning. Both groups were treated with 12 mg/kg of isotype control antibody to exclude handling effects. After conducting a repeated measures ANOVA, no significant differences were found even between 5xFAD (Isotype) and wildtypes. However, K11_IgG2a-treated mice showed a clear trend to improved hippocampus-dependent learning after 4 days when compared to the isotype-treated animals.

We further performed a pole test in order to investigate and compare motor coordination of the animals in the different treatment groups. The results of this test show a rescue of motor coordination deficits in K11_IgG2a-treated mice, whereas the 3D6_IgG2a-treated group was not affected (Additional file 10).

### 3D6_IgG2a but not K11_IgG2a treatment increases plasma Aβ level

When investigating the Aβ level in plasma of 3D6_IgG2a-treated 5xFAD mice by our total Aβ-specific ELISA, purified 3D6_IgG2b from hybridoma cell line RB96 3D6.32.2.4 (ATCC) was initially used as capture antibody. The analysis of plasma Aβ level by using the total Aβ ELISA showed no signals in 3D6_IgG2a-treated animals. This might be due to the fact that the Aβ peptides arising in the plasma of this treatment group are still in complex with the applied 3D6_IgG2a antibody, consequently preventing the interaction of 3D6_IgG2b coating antibody during ELISA analysis. In a second ELISA analysis, we used the commercially available antibody 6E10 for capturing. Again, we detected no signals in 3D6_IgG2a-treated animals, very likely due to the interference of simultaneous 3D6_IgG2a and 6E10 binding to the Aβ peptide. Finally, the application of our Aβ(X-42) ELISA, using the Aβ (17–24)-specific 4G8 as coating antibody and the Aβ(X-42)-specific 12F4 as detection antibody, enabled us to determine a considerable increase of plasma Aβ. Additional file 11 shows a highly elevated amount of Aβ(X-42) in the plasma of 3D6_IgG2a-treated animals. Please note that plasma samples were diluted 1:100 for the measurement of 3D6_IgG2a-treated animals. In contrast, Aβ(X-42) could not be detected in isotype control as well as K11_IgG2a-treated groups, even in 1:2 diluted samples. Since we obtained no ELISA signals for K11_IgG2a-treated animals in every ELISA conducted; we can exclude the possibility that interference of K11_IgG2a binding with binding of one of the other antibodies used for ELISA analyses (3D6_IgG2b, 6E10, 4G8 or 12F4) is causative for the low signals. Hence, application of K11_IgG2a does not lead to an accumulation of antibody-Aβ immune complexes in serum.

## Discussion

In spite of recent drawbacks in development of anti-Aβ vaccines, the general concept of Aβ oligomer and aggregate removal by antibodies still keeps significant promise. On our quest to develop more tailored drugs for Aβ immunotherapy, we here generated a set of highly specific monoclonal antibodies recognizing post-translational isoD7-modified Aβ. By the means of these antibodies, we confirmed the disease-related presence of isoD7-Aβ in the brain of AD patients as described before by using polyclonal isoD7-Aβ antisera [12, 13, 19].

IsoD represents a common modification of the peptide backbone, occurring preferably at asparagine but also at aspartic acid residues. The peptide sequence and three-dimensional structure affects the rate of isoD formation; hot spots are typically found if the side chain of the C-terminally adjacent amino acid is relatively small and hydrophilic and is less likely to be formed where bulky or hydrophobic residues are in this position. The most favorable C-flanking amino acids are glycine, serine, and histidine [45]. Strongly reduced rates of isoD formation are also observed at sites incorporated in rigid secondary structures. Although evidence was mentioned that the N-terminal part of Aβ is rather flexible even in the mature fibril [46] which should enable isoD formation, a recent cryo-electron microscopic analysis suggested formation of an intermolecular β-sheet within the Aβ fibril [47]. The rapid formation of isoD7 within preformed fibrils, as shown here for the first time, is rather consistent with an at least partially flexible N-terminal region of Aβ. Thus, it is very likely that isoD7-Aβ accumulates in the brain of AD patients within aggregates during peptide aging to the point of pathological brain examination post mortem. Hence, presence of the modification discerns aged Aβ from newly formed peptides, which presumably have physiological functions [48].

Moreover, we collected evidence that a mutation causing FAD is associated with an induction of isoD7 formation, thus supporting speculations from previous examinations [21–27]. To address this question, we replaced the aspartate residue in position 7 of Aβ by an asparagine. The resulting N7-Aβ peptide corresponds to the D678N-APP Tottori missense mutation leading to the development of FAD in a Japanese pedigree [28]. Because the formation of the isoD from asparagine is 10 to 100 times more accelerated compared to aspartate [29, 49], this experiment could deliver evidence for a possible function of rather isoD7-Aβ, instead of N7-Aβ, in the onset of AD. Transfection of D678N-APP in HEK293 cells leads to significantly enhanced formation of isoD7-Aβ in the cell culture supernatant. Moreover, isoD7-Aβ formation occurs very fast and can be tracked by using our ELISA systems within a few days. This observation is consistent with a putative causal function of isoD7-Aβ within the described Japanese-Tottori FAD pedigree.

In order to examine the efficacy of isoD7-Aβ antibodies in an AD mouse model, we first analyzed the presence of isoD7-Aβ in 5xFAD mice. These mice rapidly accumulate Aβ (42) in cerebrum, where amyloid plaque deposition begins at 2 months of age and increases with aging [35]. In preparation of passive immunotherapy of 5xFAD mice with our anti-isoD7-Aβ antibody, we provided evidence for the presence of isoD7-Aβ in amyloid plaques in the brains of these mice, starting before the age of 6 months and further increasing with age (see Fig. 3). At every postnatal age, isoD7-Aβ represents a fraction of the total amyloid load. However, the proportional increase of isoD7-Aβ appeared higher with increasing age and pathology (data not shown). This suggests that the isoD7 modification accumulates specifically at ages, where the phenotype, e.g., disturbances in elevated plus maze, develops. Hence, testing of a therapeutic potential of isoD7-Aβ antibodies appeared reasonable.

In order to rank the efficacy of isoD7-Aβ targeting, we directly compared our anti-isoD7-Aβ K11_IgG2a antibody to antibody 3D6_IgG2a, an IgG2a isoform of the murine version of bapineuzumab. Both antibodies show similar binding affinities to the Aβ peptide: 3–5 nM for 3D6 [43] and 4–6 nM for K11 (Table 1). Furthermore, both antibodies were recombinantly expressed as IgG2a isotype, using the same constant region. Therefore, they should have the same ability to bind and activate FcγRs. However, both antibodies differ in their epitopes: 3D6 recognizes the N-terminus of Aβ (1–40/42), whereas K11 exclusively binds the post-translationally modified variant isoD7-Aβ (Fig. 1a–c).

By applying K11_IgG2a as well as 3D6_IgG2a to 5xFAD mice, we could not only show the significant reduction of isoD7-Aβ and total Aβ level in the brain, but furthermore an improvement of cognitive deficits in different behavioral tests. Despite the fact that the epitope for the anti-isoD7-Aβ antibody K11 is much less abundant, treatment efficacy of K11_IgG2a is similar to or even better than the effects obtained by administration of 3D6_IgG2a. Moreover, in contrast to 3D6_IgG2a, K11_IgG2a treatment did not lead to an increase of plasma Aβ level. This might be due to either sequestration of 3D6_IgG2a by circulating Aβ peptides in the periphery or a general different mode of action. Because Aβ peptides are present in blood and plasma at picomolar concentrations [50], the circulating Aβ peptides capture the peripherally injected antibodies, thereby potentially reducing the amount of active antibody available for passaging the blood-brain barrier (BBB). It was already shown in other studies that the peripheral administration of monoclonal antibodies directed against non-modified Aβ enhances plasma Aβ amounts [36, 51]. In accordance with our findings, DeMattos et al. also showed that the Aβ peptides, arising in the plasma of animals after peripheral anti-Aβ antibody application, are completely bound to the administered antibodies and they hypothesized an underlying peripheral sink mechanism [51]. Besides peripheral sequestration of 3D6_IgG2a, a general difference in the mode of action might underlie the efficacy of both antibodies. Several general mechanisms are proposed how antibodies remove Aβ from the brain, among those the peripheral sink hypothesis [51, 52] and the initiation of microglial phagocytosis in the brain [41, 53]. The latter would need the antibody to enter the brain. However, beside the fact that 3D6_IgG2a enhances plasma Aβ, our analysis of 3D6-concentration in the cerebellum of 3D6_IgG2a-treated mice provides strong support for the presence of the antibody in the brain (Additional file 12). Consequently, the applied antibody crossed the BBB, although to a lower extend in comparison to K11_IgG2a, potentially due to previous sequestration by peripheral Aβ peptides.

The current results observed with isoD7-Aβ are reminiscent of results obtained with pGlu3-Aβ antibodies in several terms. Also here, not only the amount of the post-translationally modified peptide is reduced but also total Aβ level [54]. Moreover, treatment with these antibodies rescued cognitive deficits [36]. Furthermore, also isoD7-Aβ epitopes reveal an age-dependent accumulation in amyloid plaques as already demonstrated for pGlu3-Aβ [43]. In addition, the antibodies did not change the amount of Aβ in serum, whereas a strong increase in serum Aβ level was observed with antibodies binding the non-modified Aβ peptide [36, 43].

Hence, we hypothesize that passive immunotherapy by using anti-isoD7-Aβ antibodies offers several advantages:

(1) Direct targeting of aged, “non-physiological and toxic Aβ varieties”. Newly formed monomeric Aβ-peptides, which presumably have physiological functions, stay untouched. (2) No peripheral sequestration of the anti-isoD7-Aβ antibody by circulating Aβ peptides deriving from non-neuronal tissues, because there are no aged Aβ variants indicated. This might allow the reduction of effective therapeutic antibody amount and thereby decrease of antibody related side effects. (3) Aβ aggregates located in the brain may contain a certain number of isoD7-Aβ peptides, depending on their age. These epitopes are recognized by our antibody and the entire aggregates are marked for microglial phagocytosis in this way. This leads to a reduction of epitope density to be targeted by the antibody molecules and therefore fewer antibodies have to cross the blood-brain barrier. (4) If the posttranslational modification possesses a causal function in the development of AD, as it might be the case for the Japanese-Tottori mutation, the modified peptide will directly be eliminated.

Thus, targeting posttranslational modifications is certainly a different—and even might be an improved—approach than targeting non-modified Aβ.

### Limitations

One limitation should be considered. In order to achieve statistically valid results, especially for the behavioral analyses, the cohort of 5xFAD mice should have been larger than 12 animals per group. Furthermore, the results could be confirmed by its implementation in a different institution. Nevertheless, regarding the principles of the 3Rs (replacement, reduction, and refinement) in animal research, we decided to perform an exploratory treatment study with a minimal cohort size.

## Conclusion

Taken together, the present and previous studies clearly suggest that the antibody-mediated targeting of modified amyloid peptides might be favorable in several terms over previous approaches of immunotherapy. Therefore, further development of isoD7-antibodies by humanization appears warranted. Potentially, this would also open up opportunities for efficient combination therapies with approaches targeting pGlu3-Aβ in future.

## Supplementary information

**Additional file 1.** Summary of isoD7-Aβ specific antibodies obtained from murine hybridoma cells. Binding affinities of isoD7-Aβ (1–18) as well as Aβ (1–18) peptides to immobilized antibodies were determined by using Biacore 3000 at a temperature of 25 °C. Goat anti mouse IgG was immobilized to a CM5 sensor chip, followed by binding of mouse antibodies. Kinetic constants were determined by applying Aβ peptides at different concentrations and calculated from the combined set of data by using BIAevaluation software (Biacore AB).

**Additional file 2.** Immunohistochemical analysis of Aβ deposits in human brain samples by using different anti-isoD7-Aβ antibodies. Brain slices of gray and white matter from an AD patient were incubated with the antibodies A – from the first immunization experiment: K11, K16, K23 and K29 and B – from the second immunization experiment: K119, K129 and K211, followed by application of biotinylated anti-mouse IgG.

**Additional file 3.** Determination of binding affinity of K11 to isoD7-Aβ (1–18). The interaction of Aβ peptides with K11 was analyzed at a Biacore 3000 at 25 °C. Goat anti mouse IgG was immobilized on a CM5 sensor chip, followed by binding of mouse anti-isoD7-Aβ antibody K11. The binding affinity of isoD7-Aβ (1–18) was analyzed by five consecutive injections of 3 nM, 9 nM, 27 nM, 81 nM and 243 nM of the peptide. The obtained sensorgram was evaluated using the single-cycle-kinetic model getting following constants: k_a_ = 4.07·10^4^ M^− 1^ s^− 1^; k_d_ = 2.57·10^− 4^ s^− 1^ and K_D_ = 6.31 nM.

**Additional file 4.** Transmission electron microscope image of Aβ1–40 and Aβ1–42 fibrils. In each case, 10 μl of the 10 μM Aβ1–40 and Aβ1–42 fibril solutions were applied to a carbon-coated copper grid and treated with 2% (v/v) phosphotungstic acid for contrasting. Images were obtained by high-angle annular dark field scanning transmission electron microscopy using 200 kV acceleration voltage. A: Aβ (1–40)-fibrils, B: Aβ (1–42)-fibrils.

**Additional file 5.** Determination of isoD7-Aβ percentage in Aβ fibrils of different ages. Aβ (1–40) and Aβ (1–42) fibrils were incubated for 1 year at 37 °C. Samples were taken at different time points, monomerized by formic acid treatment and subsequently analyzed by isoD7-Aβ and total Aβ ELISA. A – Formation of isoD7-Aβ (1–40); B – Formation of isoD7-Aβ (1–42) within fibrillary structures.

**Additional file 6. **isoD7-Aβ formation in APP_D678N transfected HEK293 cells. HEK293 cells were transfected with wild type APP (APP_WT) and the Tottori variant APP_D678N. Samples were taken 4 and 5 days after transfection and analyzed by isoD7-Aβ and total Aβ ELISA. The error bars represent SEM. **A** – % isoD7-Aβ 4 days after transfection **B** – % isoD7-Aβ 5 days after transfection.

**Additional file 7. **Quantification of total Aβ and isoD7-Aβ peptides in T-Per fractions of 5xFAD mice brain treated with K11_IgG2a and isotype control. Three months old 5xFAD mice were treated intraperitoneally once a week with 500 μg, 150 μg K11_IgG2a or 500 μg isotype control. After 12-weeks treatment, mice were sacrificed and the left hemisphere was homogenized in T-Per buffer, followed by centrifugation. In the resulting supernatants (T-Per fractions), the amount of total Aβ (**A**) and isoD7-Aβ (**B**) was analyzed by ELISA. The error bars represent SEM.

**Additional file 8. **Elevated Plus Maze (EPM) test of mice treated weekly for 38 weeks with 12 mg/kg K11_IgG2a, 3D6_IgG2a and isotype control. Antibody-treated 5xFAD groups were compared with wildtype animals treated with 12 mg/kg isotype control. Test animals were placed with their head to the end of a defined closed arm of an elevated, plus-shaped (+) maze with two open and two enclosed arms. During the next 10 min, every movement of test animals has been recorded by a video tracking system. Arm entries are defined as presence of the complete animal (except tail) in the open arm. For statistical analysis Bonferroni’s Multiple Comparison Test was used. Sample size was at least 9 animals per group. * means *p* ≤ 0.05; ** means *p* ≤ 0.01. The error bars represent SEM.

**Additional file 9. **Fear conditioning test of mice treated weekly with 12 mg/kg K11_IgG2a, 3D6_IgG2a and isotype control. Antibody-treated 5xFAD groups were compared with wild type animals treated with 12 mg/kg isotype control. Test animals were placed in an automated Fear Conditioning System and submitted to the following procedure: pause (180 s), sound (28 s), electrical stimulus (0.7 mA for 2 s). **A – Contextual Memory:** After 24 h, test animals were again placed in the Fear Conditioning System, left there for 210 s and removed. **B – Memory of Cue:** One hour later, animals were placed back in the system, which was now covered with black walls and a black floor, in order to expose them to 180 s pause, followed by 180 s of sound (neutral stimulus). State of fear is expressed by freezing. For statistical analysis Tukey’s Multiple Comparison Test was used. Sample size was at least 8 animals per group. * means *p* ≤ 0.05. The error bars represent SEM.

**Additional file 10. **Pole test of mice treated weekly for 38 weeks with 12 mg/kg K11_IgG2a, 3D6_IgG2a and isotype control. Antibody-treated 5xFAD groups are compared with wildtype animals treated with 12 mg/kg isotype control. Animals were placed with their head directed to the top on a 50 cm high pole. Immediately after unhand, time was counted until (**A**) animals turned around (defined as every single paw is directed to the ground) and (**B**) animals reached the ground with every paw. For statistical analysis Bonferroni’s Multiple Comparison Test was used. Sample size was at least 10 animals per group. * means p ≤ 0.05; ** means p ≤ 0.01. The error bars represent SEM.

**Additional file 11.** Aβ(X-42) ELISA of plasma samples from 5xFAD animals treated weekly for 38 weeks with 12 mg/kg K11_IgG2a, 3D6_IgG2a and isotype control. Three-month-old 5xFAD mice were treated intraperitoneally once a week with 12 mg/kg K11_IgG2a, 3D6_IgG2a and isotype control. After 38 weeks of treatment, mice were sacrificed and plasma was obtained. Plasma samples from wild type, 5xFAD (isotype control) and 5xFAD (K11_IgG2a)-treated animals were diluted 1:2 and subsequently subjected to our Aβ(X-42) ELISA. In these samples, no Aβ(X-42) was detected (n.d.). Samples from 3D6_IgG2a-treated 5xFAD animals were diluted 1:100. The error bar represents SEM.

**Additional file 12.** Determination of Aβ-specific antibody concentration in cerebellum of mice treated weekly for 38 weeks with 12 mg/kg K11_IgG2a, 3D6_IgG2a and isotype control. Three months old 5xFAD mice were treated weekly by intraperitoneal injections with 12 mg/kg K11_IgG2a, 3D6_IgG2a and isotype control. After 38 weeks of treatment, mice were sacrificed; the cerebellum removed and homogenized in 300 μl ELISA Blocker + Tween with Protease Inhibitor Mix by using a Precellys homogenizer. After a centrifugation step for 15 min at 10.000 x g, followed by a second centrifugation for 30 min at 25.000 x g, supernatant was used for determination of anti Aβ-binding activity according to Frost et al., 2016. Total protein concentration was determined by using a BCA-Assay in order to calculate the antibody amounts in ng/mg total protein. The error bars represent SEM.

## Data Availability

The datasets used and/or analyzed during the current study are available from the corresponding author on reasonable request.

## Abbreviations

AD: Alzheimer’s disease
Aβ: Amyloid beta
APP: Amyloid precursor protein
ARIA: Amyloid-related imaging abnormalities
BTG: Bacterial transglutaminase
BCIP: 5-Bromo-4-chloro-3-indolyl-phosphate
isod: d-Isoaspartate
FAD: Familial AD
GdmCl: Guanidine hydrochloride
isoD: l-Isoaspartate
NBT: Nitro blue tetrazolium
3NY: Nitrotyrosin
pE: Pyroglutamate
PBST: PBS containing 0.05% Tween 20
PhosphoSer: Phosphoserine
ROI: Region of interest
SEM: Standard error of the mean
SPR: Surface plasmon resonance
TBST: TBS + 0.05% Tween 20
