# Supplementary material for: Targeting isoaspartate-modified Aβ rescues behavioral deficits in transgenic mice with Alzheimer’s disease-like pathology
Source: Alzheimers Res Ther. 2020 Nov 14;12:149. doi: 10.1186/s13195-020-00719-x (PMC7666770; doi:10.1186/s13195-020-00719-x)
Supplement: Supplementary file 1 — Additional file 1. Summary of isoD7-Aβ specific antibodies obtained from murine hybridoma cells. Binding affinities of isoD7-Aβ (1–18) as well as Aβ (1–18) peptides to immobilized antibodies were determined by using Biacore 3000 at a temperature of 25 °C. Goat anti mouse IgG was immobilized to a CM5 sensor chip, followed by binding of mouse antibodies. Kinetic constants were determined by applying Aβ peptides at different concentrations and calculated from the combined set of data by using BIAevaluation software (Biacore AB). [file 13195_2020_719_MOESM1_ESM.pdf]

Antibody	Subtype	Kd value isoD7-Aβ(1-18) [nM]	Kd value Aβ(1-18) [nM]
K11	IgG1	6.31	2700
K16	IgG1	54.8	3990
K23	IgG1	4.92	378
K29	IgG2b	136	9009
K119	IgG2a	68.5	~8000
K129	IgG1	87.7	Not detectable
K211	IgG1	208	~12100
