# Supplementary figures and images for: Targeting isoaspartate-modified Aβ rescues behavioral deficits in transgenic mice with Alzheimer’s disease-like pathology
Source: Alzheimers Res Ther. 2020 Nov 14;12:149. doi: 10.1186/s13195-020-00719-x (PMC7666770; doi:10.1186/s13195-020-00719-x)

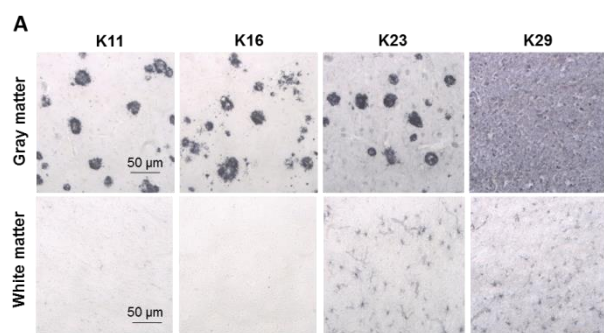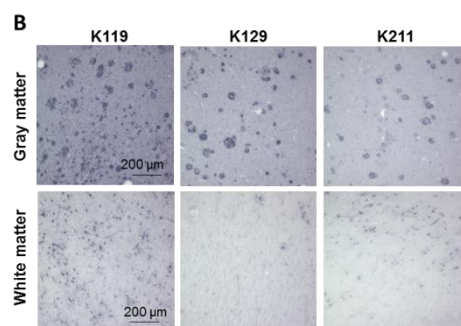

Supplement: Supplementary file 2 — Additional file 2. Immunohistochemical analysis of Aβ deposits in human brain samples by using different anti-isoD7-Aβ antibodies. Brain slices of gray and white matter from an AD patient were incubated with the antibodies A – from the first immunization experiment: K11, K16, K23 and K29 and B – from the second immunization experiment: K119, K129 and K211, followed by application of biotinylated anti-mouse IgG. [file 13195_2020_719_MOESM2_ESM.pdf]

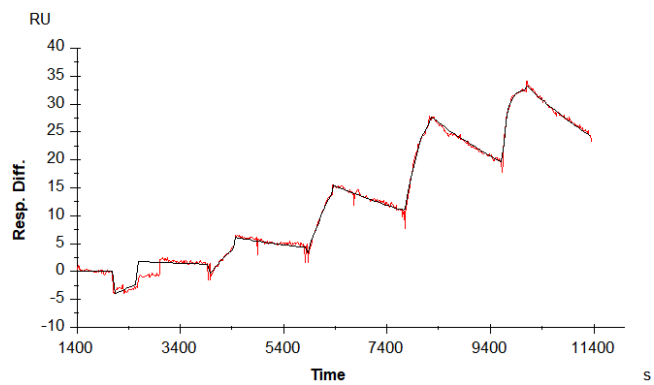

Supplement: Supplementary file 3 — Additional file 3. Determination of binding affinity of K11 to isoD7-Aβ (1–18). The interaction of Aβ peptides with K11 was analyzed at a Biacore 3000 at 25 °C. Goat anti mouse IgG was immobilized on a CM5 sensor chip, followed by binding of mouse anti-isoD7-Aβ antibody K11. The binding affinity of isoD7-Aβ (1–18) was analyzed by five consecutive injections of 3 nM, 9 nM, 27 nM, 81 nM and 243 nM of the peptide. The obtained sensorgram was evaluated using the single-cycle-kinetic model getting following constants: ka = 4.07·104 M− 1 s− 1; kd = 2.57·10− 4 s− 1 and KD = 6.31 nM. [file 13195_2020_719_MOESM3_ESM.pdf]

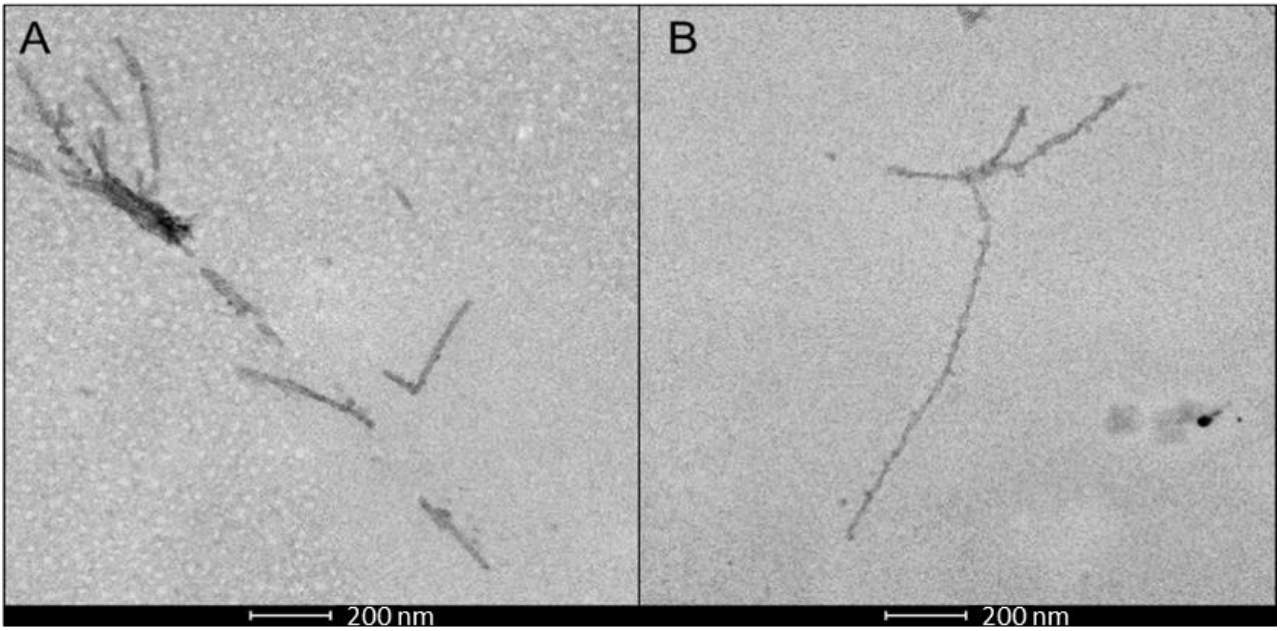

Supplement: Supplementary file 4 — Additional file 4. Transmission electron microscope image of Aβ1–40 and Aβ1–42 fibrils. In each case, 10 μl of the 10 μM Aβ1–40 and Aβ1–42 fibril solutions were applied to a carbon-coated copper grid and treated with 2% (v/v) phosphotungstic acid for contrasting. Images were obtained by high-angle annular dark field scanning transmission electron microscopy using 200 kV acceleration voltage. A: Aβ (1–40)-fibrils, B: Aβ (1–42)-fibrils. [file 13195_2020_719_MOESM4_ESM.pdf]

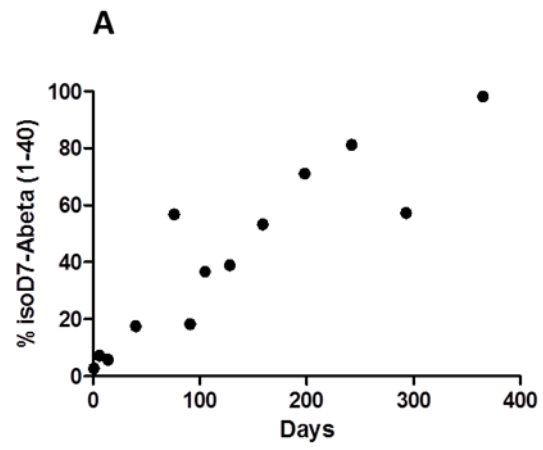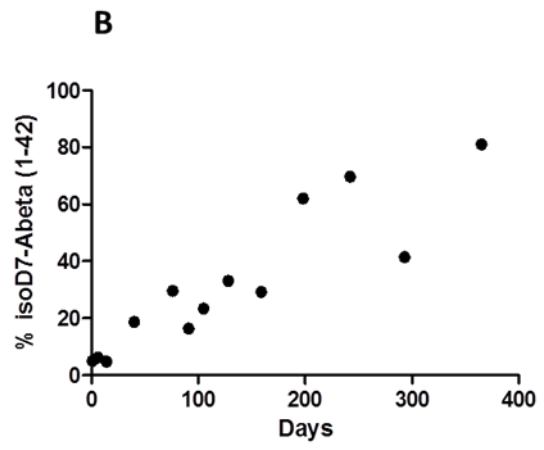

Supplement: Supplementary file 5 — Additional file 5. Determination of isoD7-Aβ percentage in Aβ fibrils of different ages. Aβ (1–40) and Aβ (1–42) fibrils were incubated for 1 year at 37 °C. Samples were taken at different time points, monomerized by formic acid treatment and subsequently analyzed by isoD7-Aβ and total Aβ ELISA. A – Formation of isoD7-Aβ (1–40); B – Formation of isoD7-Aβ (1–42) within fibrillary structures. [file 13195_2020_719_MOESM5_ESM.pdf]

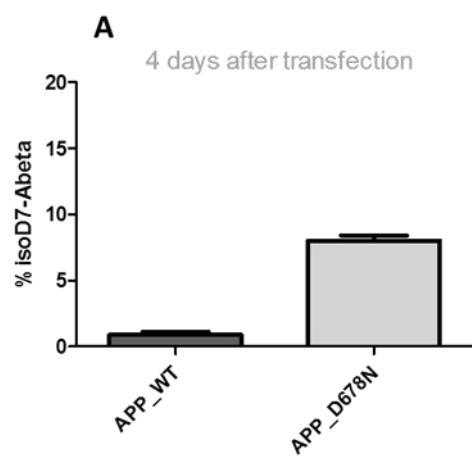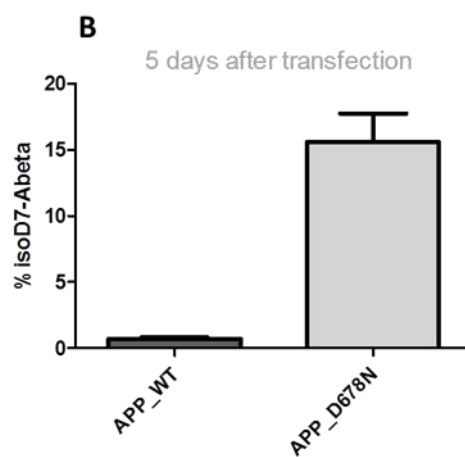

Supplement: Supplementary file 6 — Additional file 6. isoD7-Aβ formation in APP_D678N transfected HEK293 cells. HEK293 cells were transfected with wild type APP (APP_WT) and the Tottori variant APP_D678N. Samples were taken 4 and 5 days after transfection and analyzed by isoD7-Aβ and total Aβ ELISA. The error bars represent SEM. A – % isoD7-Aβ 4 days after transfection B – % isoD7-Aβ 5 days after transfection. [file 13195_2020_719_MOESM6_ESM.pdf]

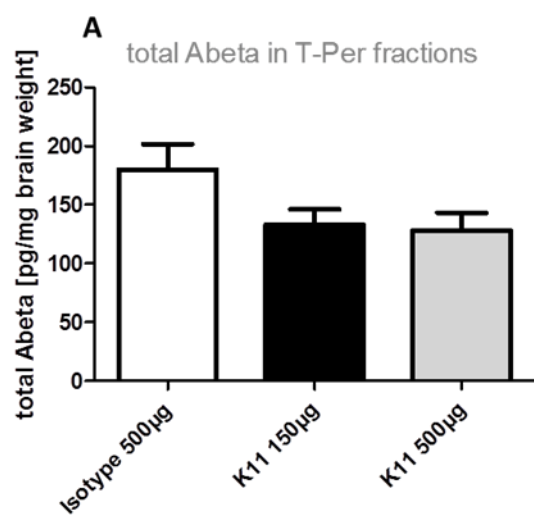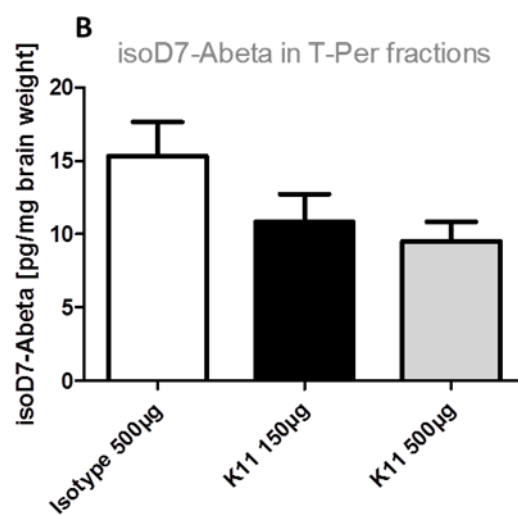

Supplement: Supplementary file 7 — Additional file 7. Quantification of total Aβ and isoD7-Aβ peptides in T-Per fractions of 5xFAD mice brain treated with K11_IgG2a and isotype control. Three months old 5xFAD mice were treated intraperitoneally once a week with 500 μg, 150 μg K11_IgG2a or 500 μg isotype control. After 12-weeks treatment, mice were sacrificed and the left hemisphere was homogenized in T-Per buffer, followed by centrifugation. In the resulting supernatants (T-Per fractions), the amount of total Aβ (A) and isoD7-Aβ (B) was analyzed by ELISA. The error bars represent SEM. [file 13195_2020_719_MOESM7_ESM.pdf]

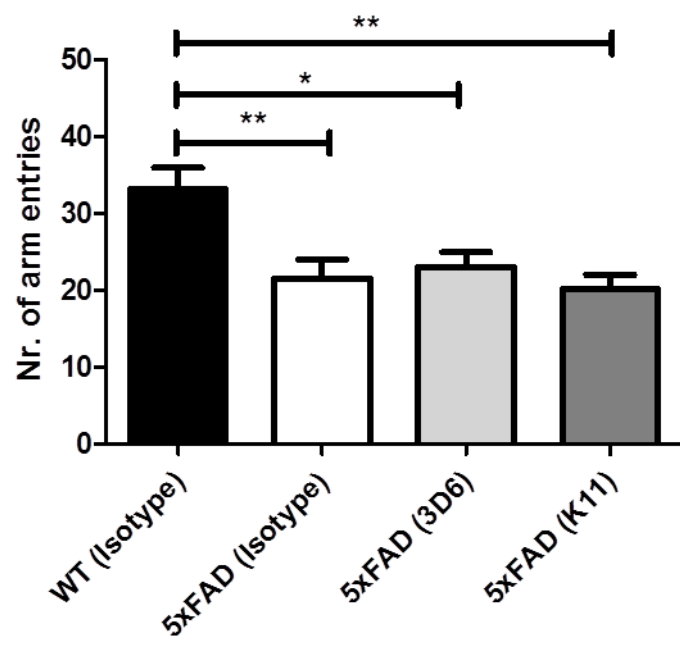

Supplement: Supplementary file 8 — Additional file 8. Elevated Plus Maze (EPM) test of mice treated weekly for 38 weeks with 12 mg/kg K11_IgG2a, 3D6_IgG2a and isotype control. Antibody-treated 5xFAD groups were compared with wildtype animals treated with 12 mg/kg isotype control. Test animals were placed with their head to the end of a defined closed arm of an elevated, plus-shaped (+) maze with two open and two enclosed arms. During the next 10 min, every movement of test animals has been recorded by a video tracking system. Arm entries are defined as presence of the complete animal (except tail) in the open arm. For statistical analysis Bonferroni’s Multiple Comparison Test was used. Sample size was at least 9 animals per group. * means p ≤ 0.05; ** means p ≤ 0.01. The error bars represent SEM. [file 13195_2020_719_MOESM8_ESM.pdf]

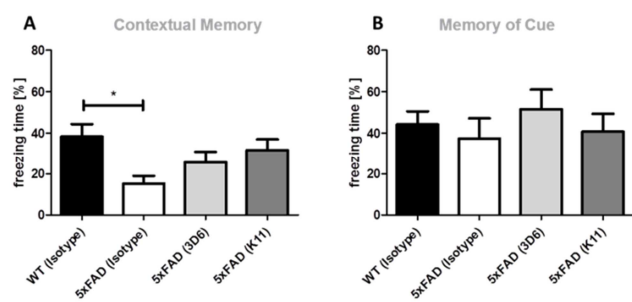

Supplement: Supplementary file 9 — Additional file 9. Fear conditioning test of mice treated weekly with 12 mg/kg K11_IgG2a, 3D6_IgG2a and isotype control. Antibody-treated 5xFAD groups were compared with wild type animals treated with 12 mg/kg isotype control. Test animals were placed in an automated Fear Conditioning System and submitted to the following procedure: pause (180 s), sound (28 s), electrical stimulus (0.7 mA for 2 s). A – Contextual Memory: After 24 h, test animals were again placed in the Fear Conditioning System, left there for 210 s and removed. B – Memory of Cue: One hour later, animals were placed back in the system, which was now covered with black walls and a black floor, in order to expose them to 180 s pause, followed by 180 s of sound (neutral stimulus). State of fear is expressed by freezing. For statistical analysis Tukey’s Multiple Comparison Test was used. Sample size was at least 8 animals per group. * means p ≤ 0.05. The error bars represent SEM. [file 13195_2020_719_MOESM9_ESM.pdf]

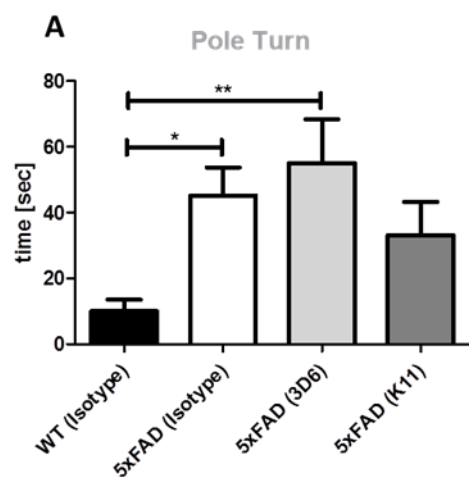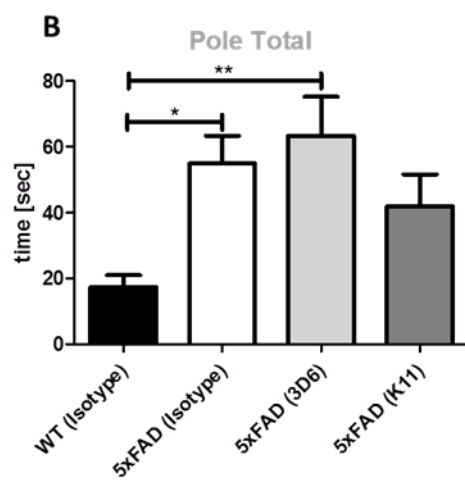

Supplement: Supplementary file 10 — Additional file 10. Pole test of mice treated weekly for 38 weeks with 12 mg/kg K11_IgG2a, 3D6_IgG2a and isotype control. Antibody-treated 5xFAD groups are compared with wildtype animals treated with 12 mg/kg isotype control. Animals were placed with their head directed to the top on a 50 cm high pole. Immediately after unhand, time was counted until (A) animals turned around (defined as every single paw is directed to the ground) and (B) animals reached the ground with every paw. For statistical analysis Bonferroni’s Multiple Comparison Test was used. Sample size was at least 10 animals per group. * means p ≤ 0.05; ** means p ≤ 0.01. The error bars represent SEM. [file 13195_2020_719_MOESM10_ESM.pdf]

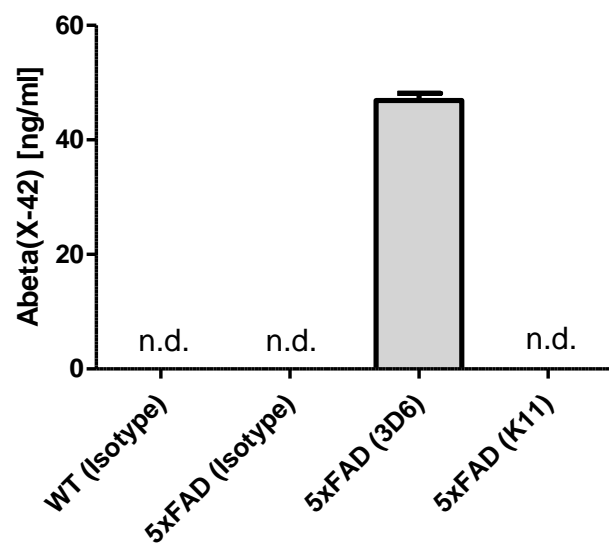

Supplement: Supplementary file 11 — Additional file 11. Aβ(X-42) ELISA of plasma samples from 5xFAD animals treated weekly for 38 weeks with 12 mg/kg K11_IgG2a, 3D6_IgG2a and isotype control. Three-month-old 5xFAD mice were treated intraperitoneally once a week with 12 mg/kg K11_IgG2a, 3D6_IgG2a and isotype control. After 38 weeks of treatment, mice were sacrificed and plasma was obtained. Plasma samples from wild type, 5xFAD (isotype control) and 5xFAD (K11_IgG2a)-treated animals were diluted 1:2 and subsequently subjected to our Aβ(X-42) ELISA. In these samples, no Aβ(X-42) was detected (n.d.). Samples from 3D6_IgG2a-treated 5xFAD animals were diluted 1:100. The error bar represents SEM. [file 13195_2020_719_MOESM11_ESM.pdf]

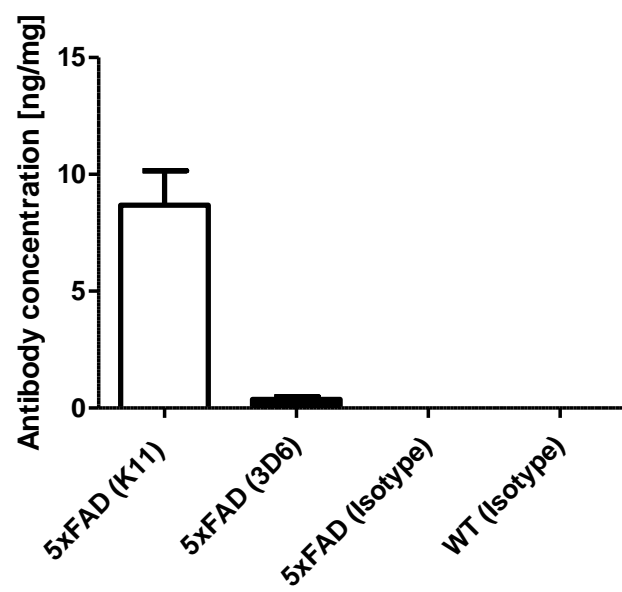

Supplement: Supplementary file 12 — Additional file 12. Determination of Aβ-specific antibody concentration in cerebellum of mice treated weekly for 38 weeks with 12 mg/kg K11_IgG2a, 3D6_IgG2a and isotype control. Three months old 5xFAD mice were treated weekly by intraperitoneal injections with 12 mg/kg K11_IgG2a, 3D6_IgG2a and isotype control. After 38 weeks of treatment, mice were sacrificed; the cerebellum removed and homogenized in 300 μl ELISA Blocker + Tween with Protease Inhibitor Mix by using a Precellys homogenizer. After a centrifugation step for 15 min at 10.000 x g, followed by a second centrifugation for 30 min at 25.000 x g, supernatant was used for determination of anti Aβ-binding activity according to Frost et al., 2016. Total protein concentration was determined by using a BCA-Assay in order to calculate the antibody amounts in ng/mg total protein. The error bars represent SEM. [file 13195_2020_719_MOESM12_ESM.pdf]
